# Reactor-Grade Non-Additive Polypropylene Architecture Defines the Physicochemical Limits of Melt Flow Index Model Transferability: An Industrial Chemometric and Machine Learning Study

**DOI:** 10.3390/polym18151880

**Published:** 2026-07-31

**Authors:** Joaquín Hernández-Fernández, Juan Lopez-Martinez

**Affiliations:** 1 Chemistry Program, Department of Natural and Exact Sciences, University of Cartagena, San Pablo Campus, Cartagena de Indias 130015, Colombia; 2 Department of Natural and Exact Sciences, Universidad de la Costa, Barranquilla 080002, Colombia; 3 Institute of Materials Technology (ITM), Universitat Politecnica de Valencia (UPV), Plaza Ferrandiz and Carbonell s/n, 03801 Alcoy, Spain

**Keywords:** polypropylene, melt flow index, machine learning, partial least squares, chemometrics, soft sensors, model transferability, polymer architecture, impact copolymers, digital twins

## Abstract

Rapid estimation of the melt flow index (MFI) is essential for the timely control of industrial polypropylene polymerization because conventional plastometer measurements require offline sampling and introduce analytical delays. This work investigates the physicochemical limits of MFI model transferability using 425 reactor-grade polypropylene production runs spanning homopolymer, random copolymer, and impact copolymer architectures. Principal component analysis (PCA), partial least squares (PLS), PCR-Ridge, Random Forest, Extra Trees, and Gradient Boosting were evaluated using within-pool, repeated five-fold, and product group validation. The latent structure, predictive performance, and dominant process descriptors were strongly architecture-dependent. Hydrogen-related variables remained central to molecular weight control, whereas comonomer descriptors, catalyst and donor variables, hydrodynamic conditions, and second reactor variables gained importance as compositional and morphological complexity increased. Nonlinear ensembles improved prediction within heterogeneous pools, but product group validation still revealed transferability losses when models crossed architecture-dependent process–structure–property domains. Repeated cross-validation and preprocessing sensitivity analyses confirmed that the principal model rankings and architecture-dependent conclusions were stable under resampling and correlation filtering. These results show that the polymer architecture defines a practical applicability boundary for industrial MFI soft sensors and supports architecture-specific or architecture-routed calibration when universal models fail cross-family validation.

## 1. Introduction

The melt flow index (MFI), also reported as the melt flow rate (MFR), is one of the most widely used quality descriptors in industrial polypropylene production. It is determined as the mass of molten polymer extruded through a standardized capillary in 10 min under prescribed temperature and load conditions, according to ISO 1133-1 and ASTM D1238 [1,2]. Although the MFI is a simple empirical measurement, its industrial relevance is considerable: it is routinely used to classify commercial grades, release production batches, select processing windows, and communicate resin specifications to end users. From a rheological perspective, the MFI provides an indirect measure of melt flowability and is inversely related to apparent melt viscosity under low-shear conditions. Consequently, it is strongly affected by the molecular weight, molecular weight distribution, chain architecture, comonomer incorporation, and phase morphology [3,4,5,6].

In polypropylene manufacturing, the interpretation of the MFI is complicated by the architectural diversity of commercial portfolios. A single industrial plant may produce homopolymers, random copolymers, and impact copolymers, each of which responds differently to reactor operating conditions. Homopolymer grades are primarily governed by hydrogen-mediated chain transfer, which regulates the molecular weight and therefore melt flow. Random copolymers introduce ethylene units along the polypropylene backbone, reducing the chain regularity, crystallinity, and melting temperature while modifying the rheological behavior [3,5,6]. Impact copolymers add a level of complexity because they are heterophasic materials consisting of a semicrystalline polypropylene matrix and a dispersed ethylene–propylene rubber phase. In these materials, the MFI is influenced not only by the matrix molecular weight but also by the rubber-phase content, phase morphology, interfacial structure, and second reactor operating conditions [4,7]. Thus, the same MFI value may arise from different molecular and morphological origins depending on the polypropylene architecture.

For industrial producers, accurate MFI prediction is important because laboratory measurements lag reactor operation. The MFI is usually measured offline or with significant sampling and analysis delays. In contrast, reactor variables such as the hydrogen feed, monomer composition, catalyst feed, donor concentration, pressure, temperature, residence time, and productivity are available continuously. This delay motivates the development of soft sensors, also referred to as virtual sensors or inferential models, which estimate difficult-to-measure quality variables from routinely measured process data [8,9]. In polypropylene production, a reliable MFI soft sensor can improve grade transition control, reduce off-spec material, support real-time quality monitoring, and assist operators in maintaining the reactor close to the target grade specification.

Several modeling strategies have been proposed for melt index prediction in polyolefin production. Linear latent variable models, particularly partial least squares (PLS) regression, have been widely used because they handle collinearity, reduce dimensionality, and provide interpretable relationships between process variables and quality variables [9,10]. Early industrial applications demonstrated that clustering and hybrid soft sensor approaches can improve the prediction of polypropylene MFI during grade transitions [8]. Regularized linear models, orthogonal least squares approaches, support vector regression, and neural network-based methods have also been explored to address nonlinearity and high collinearity in industrial datasets [11,12,13,14]. Probabilistic methods such as Gaussian process regression further extend soft sensing by providing uncertainty estimates, which are valuable for online decision-making and for detecting when a prediction falls outside the reliable operating envelope [15].

More recently, machine learning algorithms have been increasingly applied to polymer quality prediction because they can capture nonlinear interactions among process variables without requiring explicit kinetic equations. Tree-based ensemble methods, including Random Forest, Extra Trees, Gradient Boosting, and related approaches, are attractive for MFI prediction because they are relatively robust to collinearity, can model nonlinear thresholds, and provide variable importance measures. Recent studies have shown the usefulness of machine learning for predicting MFR and shear viscosity in polypropylene recyclates, optimizing industrial impact polypropylene characterization, and linking process conditions, structure, and properties in polypropylene in-reactor alloys [16,17,18]. These studies demonstrate that machine learning can improve predictive accuracy in complex polymer systems. However, improved accuracy within a dataset does not necessarily guarantee transferability across polymer architectures.

This distinction between predictive accuracy and transferability is a central unresolved issue in industrial polypropylene modeling. Most published soft sensors are validated within a single grade family, a limited operating region, or a specific grade transition dataset. Under those conditions, high predictive performance may be achieved because the calibration and validation samples belong to the same process–structure domain. However, a multigrade polypropylene portfolio is not simply a larger version of a single-grade dataset. Homopolymers, random copolymers, and impact copolymers differ in the physicochemical mechanisms that connect reactor variables to the MFI. Therefore, a model that performs well within one architecture may not remain valid when transferred to another. Transfer learning and domain adaptation methods have been proposed to address related problems in multigrade industrial processes. Still, the specific physicochemical origin of the loss of transferability in polypropylene MFI modeling remains insufficiently quantified [19,20,21,22,23].

Two divergent views can be identified in the current literature. The first view is algorithmic: model transferability can be improved primarily by using more flexible methods, such as nonlinear regression, ensemble learning, Gaussian process models, or deep learning-based soft sensors. According to this view, poor transferability is primarily a statistical limitation caused by nonlinearity, small sample sizes, or insufficient model capacity [15,16,17,18,24]. The second view is physicochemical: transferability is limited because different polypropylene architectures occupy different process–structure–property domains. From this perspective, model failure across grades is not only a mathematical problem but also a material science problem. The same process variable may have different physical meanings depending on whether the product is a homopolymer, a random copolymer, or an impact copolymer. For example, the ethylene content acts as a chain irregularity descriptor in random copolymers but as a rubber-phase-related descriptor in impact copolymers; similarly, hydrogen primarily regulates the molecular weight but may interact differently with the catalyst activity, comonomer incorporation, and phase development across architectures [3,4,5,6,7].

Despite substantial progress in soft sensor design, several limitations remain. First, many studies report within-grade or within-family prediction accuracy but do not explicitly quantify cross-architecture transferability. Second, latent variable descriptions are often interpreted globally, even though the dominant process variables and their loadings may rotate across architectures. Global prediction metrics frequently evaluate third- and fourth-order nonlinear machine learning models. At the same time, less attention is given to whether their learned relationships remain physically meaningful and transferable across product families. Fourth, the role of the polymer architecture is often treated as a categorical or operational label rather than a mechanistic descriptor that links reactor variables, molecular structure, morphology, and melt rheology.

The present study addresses these limitations by investigating the transferability of MFI prediction models across an industrial polypropylene portfolio containing homopolymer, random copolymer, and impact copolymer production data. The work combines principal component analysis, PLS regression, regularized linear modeling, and tree-based machine learning algorithms to evaluate three connected questions: (i) whether different polypropylene architectures occupy distinct latent statistical domains; (ii) whether linear latent variable models retain predictive accuracy when heterogeneous architectures are merged into a universal calibration space; and (iii) whether nonlinear machine learning models improve prediction without fully eliminating architecture-dependent transferability loss.

The main hypothesis is that the polymer architecture imposes physicochemical limits on model transferability. Under this hypothesis, the MFI should not be interpreted only as a function of the molecular weight or hydrogen concentration, but as the macroscopic result of an architecture-dependent structure–property cascade involving the molecular weight, molecular weight distribution, ethylene incorporation, crystallinity, rubber-phase morphology, and melt rheology. The principal conclusion anticipated by this framework is that architecture-aware models provide a more physically meaningful and statistically robust strategy than architecture-blind universal calibration approaches for industrial polypropylene MFI prediction.

## 2. Materials and Methods

### 2.1. Industrial Polypropylene Plant and Dataset Structure

The industrial database comprised 425 production runs collected during routine operation of a commercial gas-phase polypropylene plant. The portfolio covered 41 commercial grades representative of the three major polypropylene architectures produced in industrial practice: homopolymers, random copolymers, and heterophasic impact copolymers. The database was deliberately organized into five modeling pools: the homopolymer H-05H82 pool, the random copolymer R-MF(2–5) pool, the impact copolymer pool, the combined random + impact copolymer pool, and the full H+R+I portfolio. This organization was designed to evaluate both within-architecture prediction and cross-family model transferability.

It is important to emphasize that the present study does not investigate commercial polypropylene pellets. Instead, all samples correspond to reactor-grade polypropylene powder collected directly after gas-phase polymerization and before any pelletization, extrusion, stabilization, antioxidant addition, nucleating agent incorporation, or commercial formulation. Consequently, the measured melt flow index reflects the intrinsic rheological response arising exclusively from the polymerization process and the resulting molecular architecture. This distinction is fundamental because downstream extrusion and additive incorporation may substantially modify the rheological behavior of commercial polypropylene without altering the polymerization mechanisms investigated in the present work.

**Figure 1:**
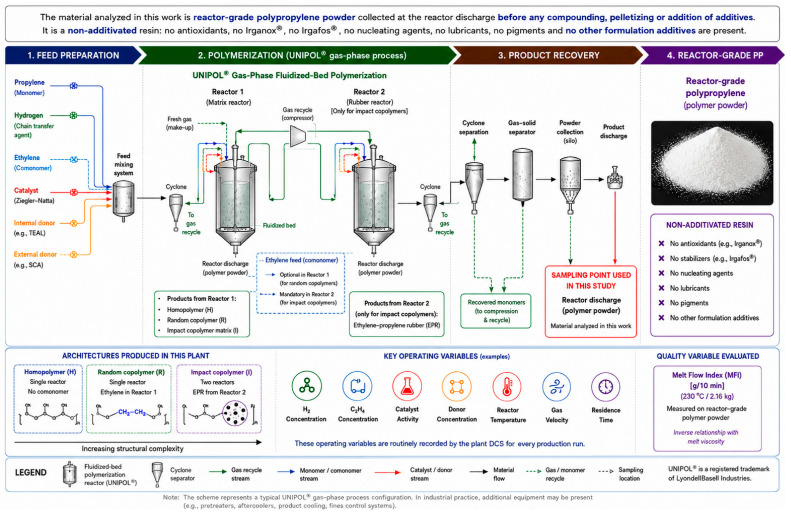
Industrial production route and sampling strategy for reactor-grade polypropylene before pelletization and additive incorporation.

Depending on the modeling pool, between 26 and 40 independent process variables were available before filtering and harmonization. These variables included catalyst-related descriptors, hydrogen and monomer/comonomer composition, cocatalyst and donor ratios, reactor pressure, production rate, bed inventory, fluidization descriptors, residence time-related variables, and reactor temperatures. The final harmonized modeling matrix used for the comparative analyses retained the common descriptors available across the corresponding pool. In contrast, architecture-specific descriptors were retained when required for impact copolymer and combined pool modeling. The final dataset composition is reported in Table S1, whereas the statistical structure of the industrial portfolio is summarized in Figure 1.

### 2.2. Description of Polypropylene Architectures

The five operating pools used in this study are summarized in Table S1. The portfolio was assembled to span the three principal architectural families of isotactic polypropylene: homopolymer, random copolymer, and heterophasic impact copolymer. Two combined pools were also evaluated: the random + impact pool, representing an intermediate multi-architecture calibration space, and the full H+R+I pool, representing the universal portfolio used to test whether a single predictive model can be transferred across polypropylene architectures.

The H-05H82 pool comprised 27 production runs from one homopolymer family. This pool represents the baseline architecture of isotactic polypropylene produced in a gas-phase reactor without comonomer feed. In this family, the MFI is primarily regulated by hydrogen-mediated chain transfer, although the catalyst feed, cocatalyst/donor balance, reactor temperature, residence time, and fluidization regime can modulate the final response [3,6,8,24]. From a chemometric perspective, this pool is challenging due to its limited sample size and high variable-to-sample ratio, which increase the risk of overparameterization.

The R-MF(2–5) pool comprised 21 production runs from random copolymer grades. Random copolymers are poly(propylene-co-ethylene) materials in which ethylene units are incorporated along the polypropylene backbone. This comonomer incorporation reduces the chain regularity, crystallinity, melting temperature, and modulus relative to homopolymer polypropylene [3,5,6]. In this architecture, the MFI is controlled by a combined molecular weight/comonomer axis: hydrogen-related variables regulate the molecular weight, whereas ethylene-related descriptors modulate chain irregularity and crystallization behavior.

The impact copolymer pool comprised 110 production runs from heterophasic impact copolymer grades. Impact copolymers are two-phase materials composed of a semicrystalline polypropylene matrix and a dispersed ethylene–propylene rubber phase. Their rheological and mechanical behavior is therefore influenced not only by the matrix molecular weight but also by the rubber-phase content, phase morphology, interfacial structure, and second reactor operating variables [4,7,17]. This pool represents the most structurally complex single-architecture family in the dataset.

The R+I pool comprised 147 runs combining random and impact copolymers. This pool was included to evaluate an intermediate universal model in which ethylene-containing materials are merged despite having different structural origins of ethylene incorporation. Finally, the H+R+I pool contained all 425 runs and was used as the full-portfolio calibration space. This pool was used to evaluate whether a single architecture-blind model can describe the complete industrial polypropylene portfolio.

#### 2.2.1. Homopolymer Grades

Homopolymer isotactic polypropylene grades are produced without comonomer feed. In these grades, hydrogen is the main small-molecule chain transfer agent and therefore the principal manipulated variable for controlling the molecular weight and MFI [8,24]. Under ideal open-loop polymerization conditions, increasing hydrogen availability promotes chain transfer, decreases the average chain length, and increases the MFI. In industrial closed-loop operation, however, the empirical relationship between hydrogen and the MFI may be altered by feedback trim, as the hydrogen feed is adjusted in response to laboratory MFI deviations. This distinction is important when interpreting process data: the kinetic role of hydrogen and its statistical loading in an industrial database are not necessarily equivalent.

In this study, the H-05H82 pool was used as the simplest architecture-restricted benchmark. Its purpose was to evaluate whether linear latent variable modeling can estimate reactor-grade MFI for real-time process monitoring when comonomer incorporation and heterophasic morphology are absent. 

**Figure 2:**
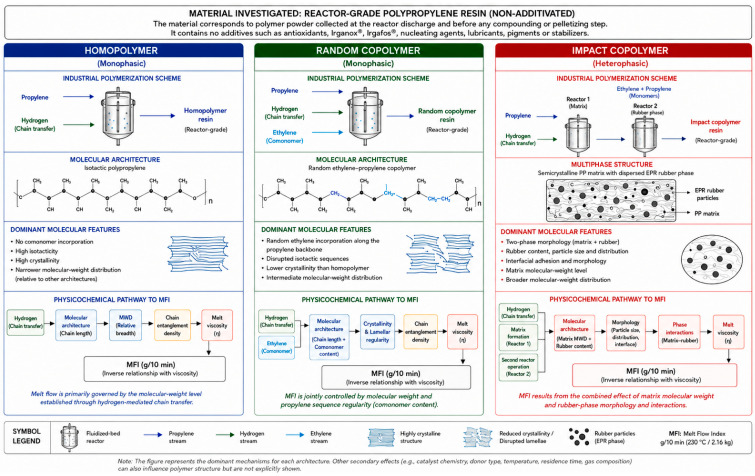
Physicochemical mechanisms governing the generation of the melt flow index (MFI) in the three architectures of reactor-grade non-additive polypropylene.

Although homopolymer polypropylene represents the simplest architecture included in the present study, the molecular origin of its melt flow behavior is still governed by several interconnected physicochemical mechanisms, as summarized in Figure 2. In gas-phase Ziegler–Natta polymerization, hydrogen acts as the principal chain transfer agent, thereby reducing the average molecular weight by promoting chain termination. As the molecular weight decreases, the chain entanglement density decreases, resulting in lower melt viscosity and, consequently, higher MFI values. Nevertheless, the molecular weight alone does not fully determine the industrial melt flow response. Variations in catalyst productivity, donor efficiency, active site distribution, reactor temperature, gas composition, residence time, and fluidization conditions also modify the molecular weight distribution and stereoregularity of the polymer chains. These secondary effects influence the crystallization kinetics and rheological behavior, introducing additional variability into the industrial dataset. Consequently, even within a chemically simple homopolymer architecture, the MFI should be interpreted as the macroscopic manifestation of multiple coupled reactor-scale and molecular-scale phenomena rather than as the direct consequence of hydrogen concentration alone.

#### 2.2.2. Random Copolymer Grades

Random polypropylene copolymers are produced in a single gas-phase reactor with an additional ethylene feed. Ethylene insertion interrupts isotactic propylene sequences and modifies the crystallinity, melting temperature, and flow-induced crystallization behavior [3,5,6]. In contrast to homopolymers, the MFI of random copolymers is governed not only by molecular weight control but also by comonomer incorporation. Therefore, hydrogen-related descriptors and ethylene-related descriptors must be considered together when interpreting the random copolymer modeling space.

**Figure 3:**
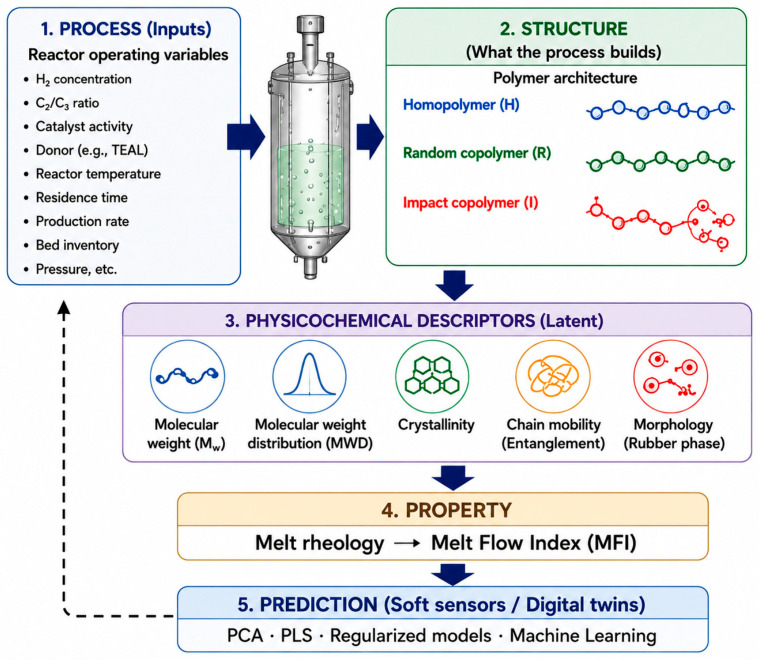
Process–structure–property–prediction framework.

**Figure 4:**
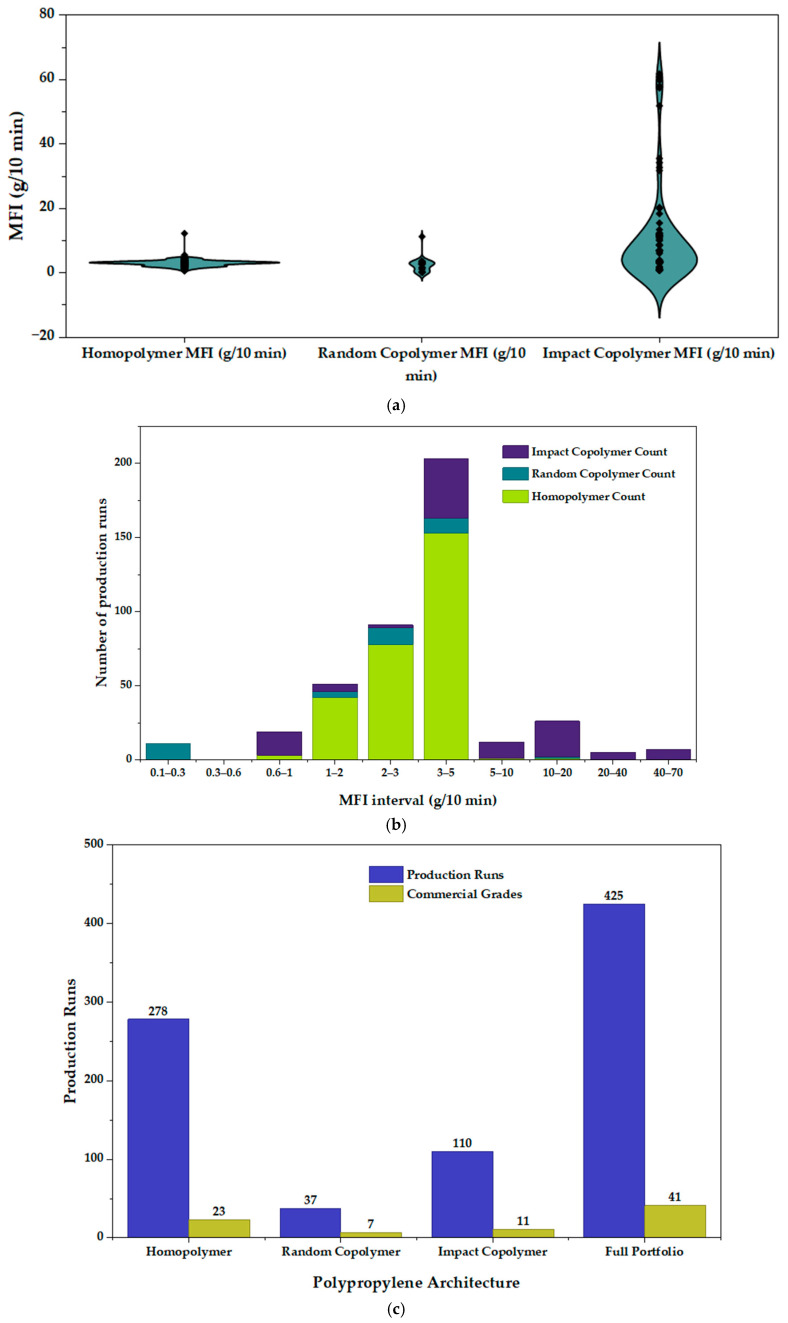
Statistical structure of the industrial polypropylene portfolio used for MFI modeling. (**a**) Distribution of melt flow index values across homopolymer, random copolymer, and impact copolymer architectures. (**b**) Frequency distribution of MFI values using logarithmically spaced intervals, showing the architecture-specific contribution to each flow range. (**c**) Composition of the industrial dataset in terms of production runs and commercial grades for each polypropylene architecture. MFI values are expressed in g/10 min.

**Figure 5:**
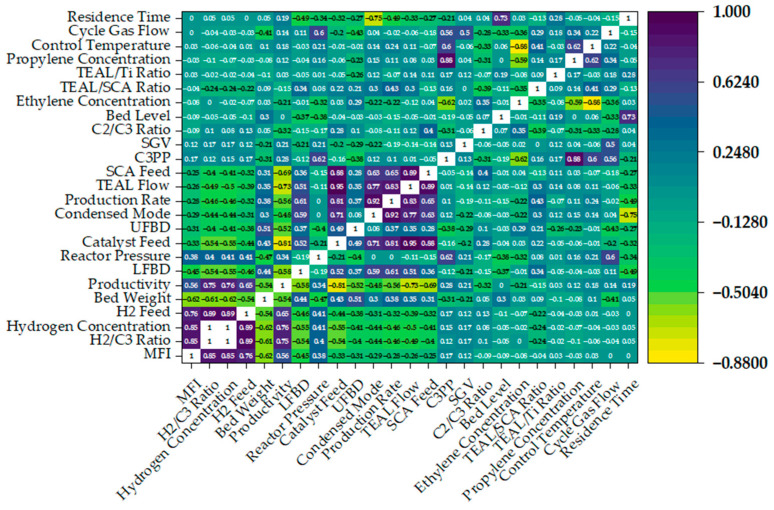
Pearson correlation matrix of industrial process variables and melt flow index. The heatmap reports Pearson correlation coefficients among MFI and the industrial process variables used for chemometric and machine learning modeling. Warm colors represent positive correlations, whereas cool colors represent negative correlations. The matrix identifies the principal kinetic, compositional, and hydrodynamic relationships present in the industrial database.

**Figure 6:**
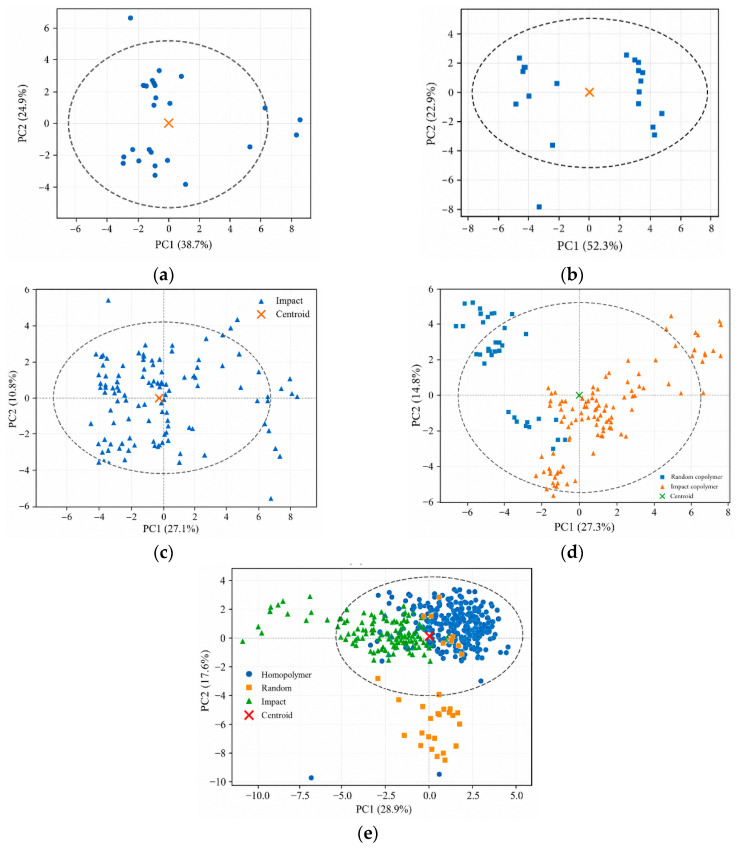
Principal component analysis score plots of the industrial polypropylene datasets. (**a**) Homopolymer H-05H82 dataset, (**b**) random copolymer R-MF(2–5) dataset, (**c**) impact copolymer dataset, (**d**) combined random and impact copolymer dataset, and (**e**) full polypropylene portfolio comprising homopolymer, random copolymer, and impact copolymer production runs. Scores are plotted on the first two principal components, with the percentage of explained variance indicated on each axis. The dashed ellipse represents the score space confidence region, and the centroid marks the center of each PCA domain.

**Figure 7:**
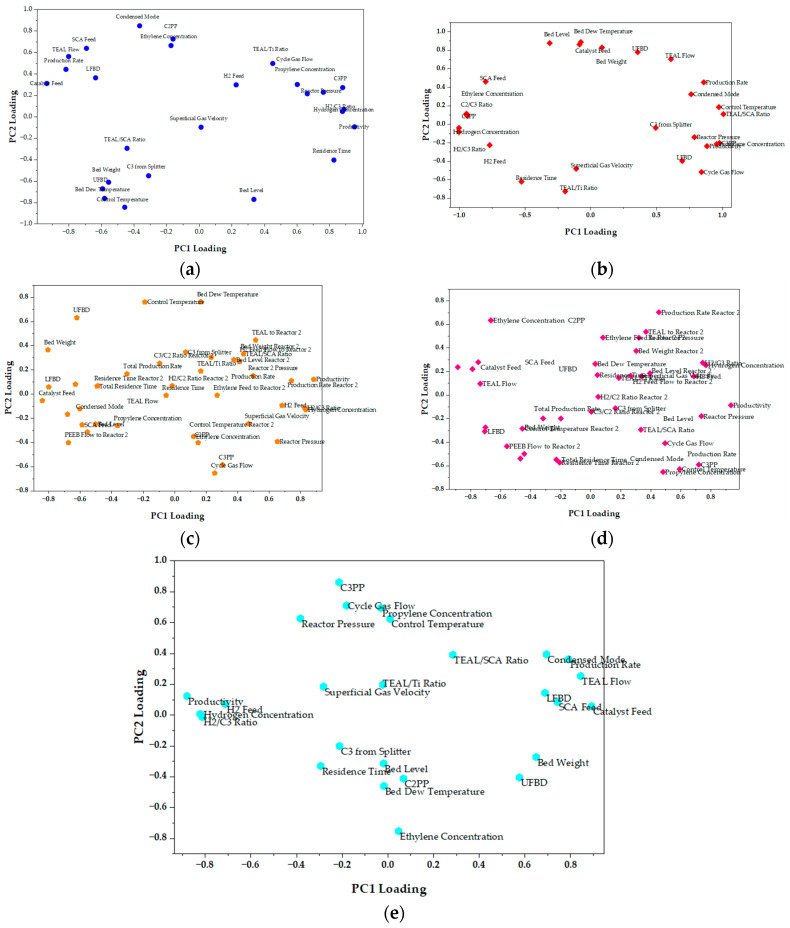
PCA loading plots showing the contribution of the operational variables to each industrial dataset. (**a**) Homopolymer H-05H82 dataset, (**b**) random copolymer R-MF(2–5) dataset, (**c**) impact copolymer dataset, (**d**) combined random and impact copolymer dataset, and (**e**) full polypropylene portfolio. Variables are projected onto their PC1 and PC2 loadings, thereby identifying the process descriptors that dominate each latent statistical domain.

**Figure 8:**
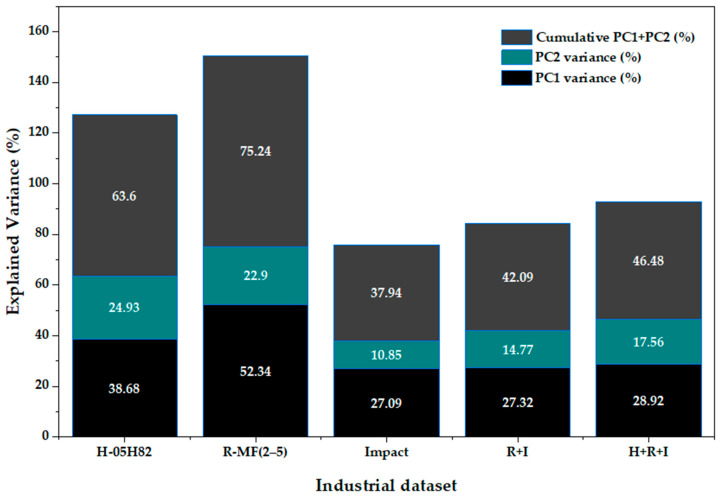
PCA explained variance and latent space compressibility across polypropylene architectures. The stacked bars show the individual contributions of PC1 and PC2 for each industrial dataset, while the labels indicate the cumulative explained variance. The decrease in PC1+PC2 variance from single-architecture datasets to impact-rich and full-portfolio datasets evidences the reduced latent space compressibility associated with increasing architectural complexity.

**Figure 9:**
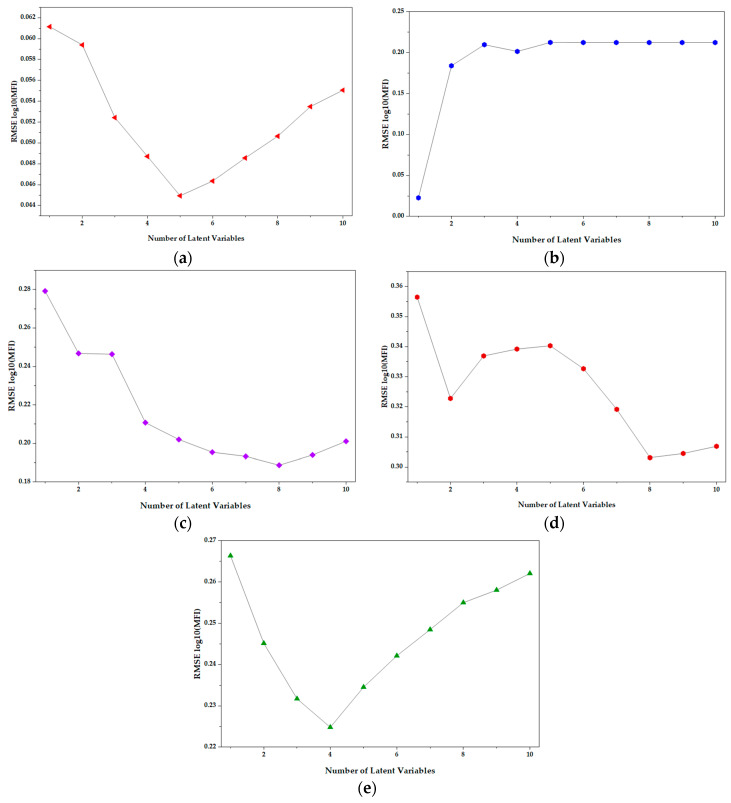
Optimization of the number of latent variables used in the PLS models for MFI prediction: (**a**) homopolymer H-05H82 dataset; (**b**) random copolymer R-MF(2–5) dataset; (**c**) impact copolymer dataset; (**d**) combined random and impact copolymer (R+I) dataset; and (**e**) full polypropylene portfolio comprising homopolymer, random copolymer, and impact copolymer datasets (H+R+I). Cross-validated RMSE values are plotted as a function of the number of latent variables. The selected number of components corresponds to the minimum cross-validated RMSE, representing the optimal balance between predictive accuracy and model complexity.

The R-MF(2–5) pool was used to evaluate whether a comonomer-containing but still monophasic architecture can be described by a compact latent structure. The PCA results for this pool are presented in Figure 3, Figure 4 and Figure 5, and the optimized PLS model is reported in Figure 6, Figure 7, Figure 8 and Figure 9.

In random polypropylene copolymers, the incorporation of ethylene introduces an additional physicochemical dimension absent in homopolymers. Ethylene units are randomly distributed along the polypropylene backbone, interrupting isotactic propylene sequences and decreasing lamellar regularity. This molecular modification reduces the crystallinity, lowers the melting temperature, alters crystal perfection, and changes chain mobility during melt deformation. As a consequence, the final MFI no longer depends exclusively on molecular weight reduction via hydrogen-mediated chain transfer. Instead, it reflects the combined influence of the molecular weight and comonomer incorporation. This dual control mechanism explains why hydrogen-related variables remain important while ethylene descriptors simultaneously acquire predictive relevance in chemometric and machine learning models.

#### 2.2.3. Impact Copolymer Grades

Heterophasic impact polypropylene grades are produced through a two-stage architecture in which a polypropylene matrix is generated first, and an ethylene–propylene rubber phase is subsequently incorporated under a different reactor environment. This architecture introduces descriptors that are absent in homopolymer and random copolymer grades, including second reactor productivity, second reactor gas composition, residence time, bed behavior, and variables related to rubber-phase development [4,7,17].

**Figure 10:**
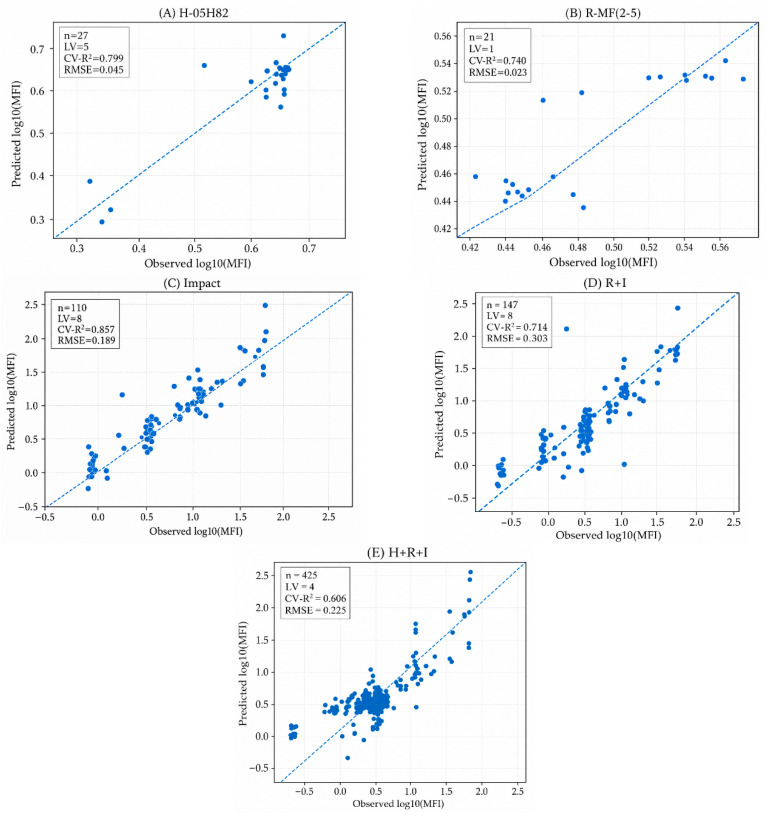
Cross-validated PLS predicted-versus-observed melt flow index. Predicted and observed log10(MFI) values are compared for (**A**) homopolymer H-05H82, (**B**) random copolymer R-MF(2–5), (**C**) impact copolymer, (**D**) random + impact copolymers, and (**E**) the full polypropylene portfolio. The dashed diagonal represents the 1:1 reference line. Insets summarize the sample size, selected latent variables, CV-R^2^, RMSE, and MAE.

**Figure 11:**
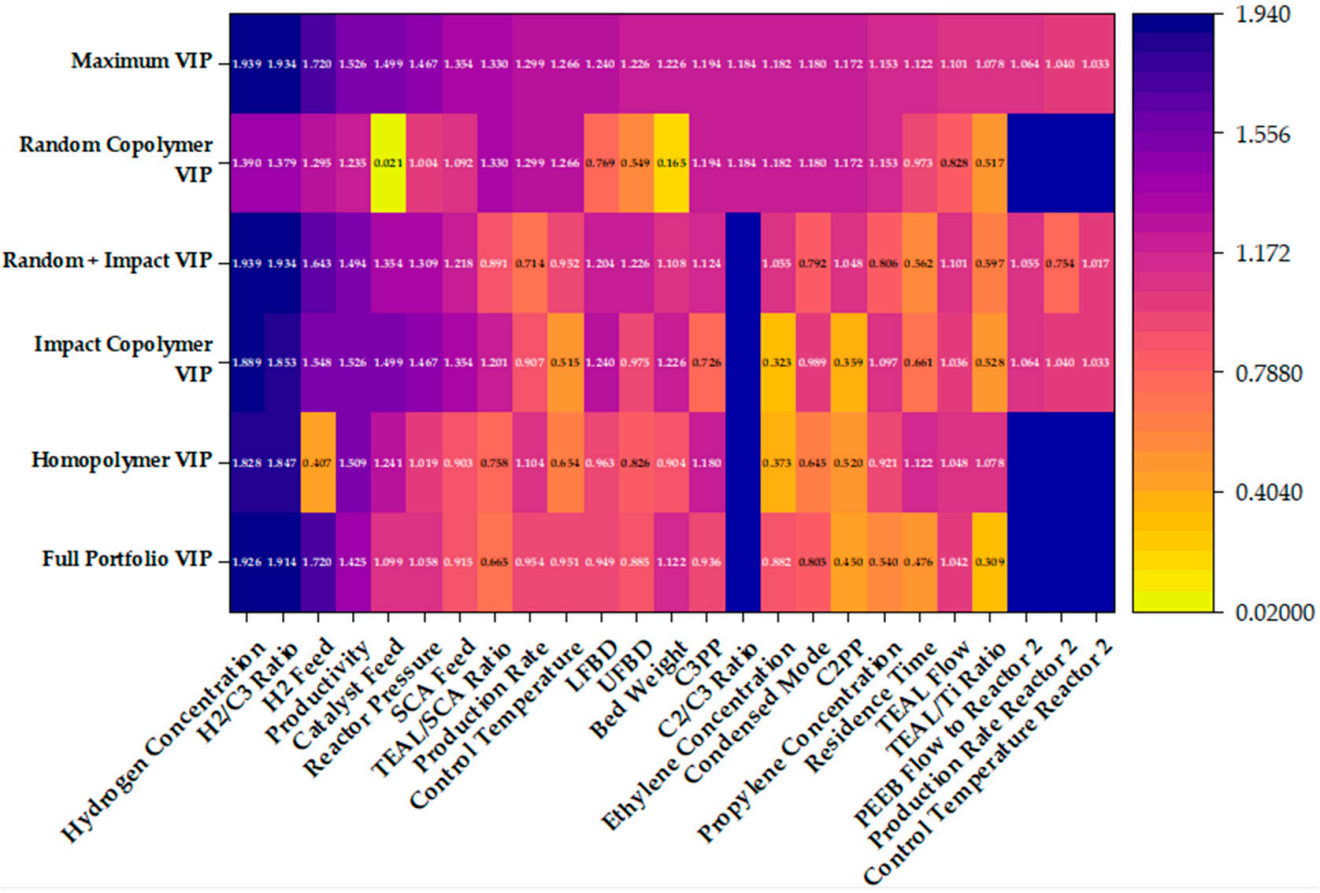
Variable Importance in Projection scores obtained from the PLS models across the industrial polypropylene datasets. VIP scores quantify the relative contribution of each process descriptor to the prediction of log10(MFI). Variables with VIP ≥ 1 were considered influential contributors to the corresponding PLS model. Blank cells indicate variables that were unavailable or not applicable to a given dataset.

The impact pool was included to evaluate whether a structurally complex but internally coherent architecture can still be modeled effectively. Because all samples in this pool belong to the same heterophasic family, the model does not need to transfer across fundamentally different polypropylene architectures. This distinction is important for interpreting the difference between within-pool prediction, reported in Figure 7, Figure 9, Figure 10 and Figure 11.

Impact copolymers exhibit the highest structural complexity among the investigated architectures because they are produced through sequential polymerization stages. The first reactor generates a semicrystalline polypropylene matrix, whereas the second reactor produces an ethylene–propylene rubber phase dispersed throughout the matrix. Consequently, the melt flow behavior depends simultaneously on the matrix molecular weight, rubber content, particle morphology, interfacial adhesion, and phase distribution. Unlike homopolymers and random copolymers, in which the molecular architecture is essentially monophasic, impact copolymers contain multiple structural levels that independently contribute to rheological performance. Therefore, process variables associated with the second reactor are expected to become statistically relevant, even when their direct influence on the molecular weight is limited.

### 2.3. Industrial Database Construction

Each production run corresponded to a steady-state interval in industrial production. Process variables were extracted from the plant distributed control system and aggregated into run-level descriptors. The aggregation window was selected to be centered on steady-state operation to minimize the contributions of grade transition transients, start-up effects, and non-representative operating fluctuations. Each row in the final database contained process descriptors, the corresponding laboratory MFI value, and metadata identifying the commercial grade and polymer architecture.

The database was partitioned into five modeling pools: H-05H82, R-MF(2–5), impact, R+I, and H+R+I. These pools were analyzed independently for architecture-restricted modeling and then compared using common validation protocols. The final portfolio composition is summarized in Table S1 and Figure 1.

### 2.4. Melt Flow Index Determination and Response Transformation

The melt flow index was determined according to standardized melt flow testing procedures for thermoplastics [1,2]. For each production run, the laboratory MFI value was assigned to the corresponding steady-state process window. When duplicate measurements were available, the mean value was retained as the run-level response.

Because MFI values spanned a broad industrial range, the response variable used for chemometric and machine learning regression was log10(MFI). This transformation was applied to reduce heteroscedasticity and to avoid over-weighting high-flow grades during model calibration. All reported regression metrics, including CV-R^2^, RMSE, and MAE, were therefore calculated on the log10(MFI) scale unless otherwise specified. The distribution of the MFI across architectures is shown in Figure 1.

### 2.5. Process Variables

The process descriptors retained for chemometric and machine learning analysis were grouped into four families, as summarized in Table S2. Catalyst-related descriptors included the catalyst feed, TEAL flow, TEAL/SCA ratio, SCA feed, and donor-related ratios. These variables describe active site concentration, cocatalyst availability, donor selectivity, and catalyst productivity.

Monomer and comonomer descriptors included propylene-related variables, ethylene-related variables, the C2/C3 ratio, and C2PP and C3PP. These variables describe the gas-phase composition and are particularly relevant for random and impact copolymer pools.

Molecular weight control descriptors included the hydrogen concentration, H2/C3 ratio, and H2 feed. These descriptors are associated with chain transfer control and are expected to be strongly related to the MFI, although their empirical correlation may be influenced by closed-loop process control.

**Figure 12:**
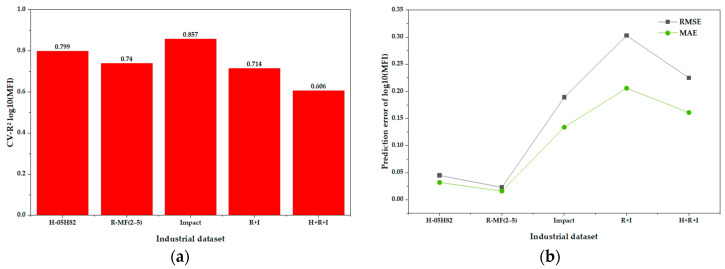
Comparative performance of the PLS models across the industrial polypropylene datasets. (**a**) Cross-validated coefficient of determination for log10(MFI) prediction. (**b**) Cross-validated prediction errors expressed as RMSE and MAE of log10(MFI).

**Figure 13:**
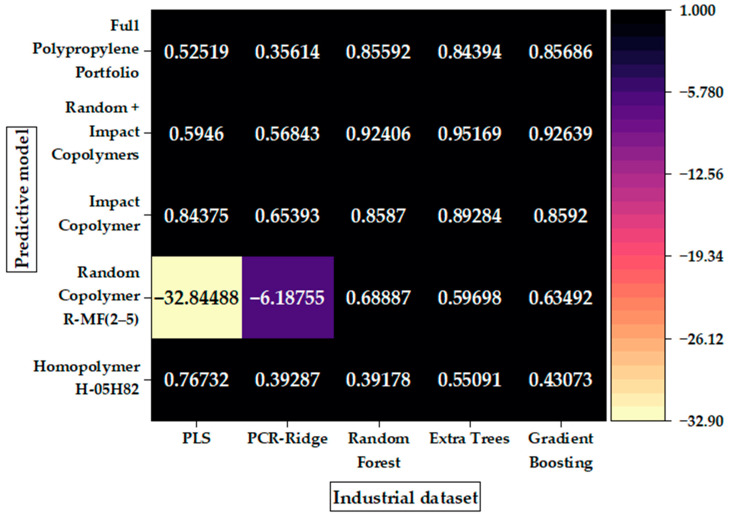
Machine learning benchmarking for log10(MFI) prediction across the industrial polypropylene datasets. The heatmap reports cross-validated R^2^ values obtained for PLS, PCR-Ridge, Random Forest, Extra Trees, and Gradient Boosting models. Negative CV-R^2^ values indicate models that performed worse than the mean response baseline under the corresponding validation protocol.

Thermal, hydrodynamic, and productivity descriptors included the control temperature, reactor pressure, cycle gas flow, bed level, bed weight, UFBD, LFBD, condensed mode operation, residence time, productivity, production rate, and reactor-2-specific production variables when applicable. These variables describe heat removal, fluidization, residence time distribution, and architecture-specific reactor operation. Their importance was evaluated using Pearson correlation analysis, PCA loadings, PLS VIP scores, and tree-based feature importance, as reported in Figure 2, Figure 4, Figure 8, Figure 12 and Figure 13.

### 2.6. Dataset Preprocessing

The raw industrial database was preprocessed before multivariate modeling. First, duplicate laboratory MFI values were averaged when available, and observations with inconsistent laboratory flags were inspected before modeling. Second, rows corresponding to non-steady-state or incomplete operating conditions were excluded. Third, missing values were treated according to the variable type and pool coverage; variables with insufficient coverage in a given pool were excluded from that pool rather than imputed across incompatible architectures.

Fourth, each process variable was mean-centered and scaled to unit variance within the corresponding modeling pool. This scaling was required because the descriptors were expressed in different units and magnitudes. Without scaling, high-magnitude process variables would dominate the PCA, PLS, and distance-sensitive model components. Fifth, strongly redundant variables were inspected using Pearson correlation analysis. Variable pairs with an absolute Pearson correlation coefficient greater than 0.97 were reviewed to avoid redundant descriptors and excessive collinearity. When two variables represented the same physical signal, the more interpretable or more consistently available descriptor was retained. Sixth, log10(MFI) was used as the response variable for all regression models, as described in Section 2.4.

The final preprocessed matrices were used consistently for PCA, PLS, PCR-Ridge, Random Forest, Extra Trees, and Gradient Boosting. The correlation structure of the processed industrial descriptors is shown in Figure 2.

The effectiveness of the logarithmic transformation was further verified by comparing residual dispersion before and after applying the log10(MFI) transformation. As illustrated in Supplementary Figure S3, the logarithmic transformation substantially reduces heteroscedasticity across the entire industrial operating range, yielding a more homogeneous residual distribution and improving compliance with the assumptions underlying chemometric calibration. Consequently, the transformation enhances model stability without altering the underlying physicochemical relationships governing melt flow generation. Since correlation-based variable filtering may influence latent space organization, an additional sensitivity analysis was conducted by comparing model performance before and after removing highly correlated predictors. The results presented in Supplementary Figure S4 demonstrate that the predictive performance remains largely unchanged regardless of the preprocessing strategy, confirming that the adopted correlation filtering primarily removes redundant information while preserving the dominant physicochemical descriptors governing melt flow behavior. Therefore, the observed architecture-dependent differences cannot be attributed to preprocessing artifacts.

### 2.7. Principal Component Analysis

Principal component analysis was applied independently to each modeling pool using mean-centered and variance-scaled process variables. PCA was used as an unsupervised diagnostic tool to evaluate the latent statistical structure of each polypropylene architecture. The analysis focused on three outputs: score space geometry, loading vector orientation, and explained variance. This framework follows established chemometric practice for interpreting multivariate process data [9,25].

The first two principal components were used for visual comparison across datasets. PCA score plots were used to evaluate whether production runs occupied compact or architecture-dependent latent domains, as reported in Figure 3. PCA loading plots were used to identify the process variables that define each latent direction, as reported in Figure 4. The variance explained by PC1 and PC2 was used to quantify latent space compressibility across architectures, as reported in Table 1 and Figure 5.

The PCA models were fitted separately for H-05H82, R-MF(2–5), impact, R+I, and H+R+I pools. This independent fitting strategy was selected because the objective was not to force all architectures into a common PCA model, but to determine whether each architecture possessed a distinct latent structure.

The optimized hyperparameters selected for each tree-based algorithm are summarized in Supplementary Table S6. Although hyperparameter optimization was performed independently for each predictive model, preliminary evaluations using alternative optimization strategies produced comparable model rankings. This observation suggests that the superiority of the best-performing algorithms primarily stems from their intrinsic ability to capture architecture-dependent nonlinearities rather than from any particular hyperparameter configuration.

### 2.8. Partial Least Squares Regression

Partial least squares regression was used as the primary linear latent variable baseline for MFI prediction. PLS was selected because it is widely used in chemometrics and industrial soft sensing for its ability to handle collinearity and relate process variables to a response variable using a reduced number of latent variables [10,26]. The SIMPLS algorithm was used to estimate the PLS models [27].

For each modeling pool, the process variable matrix was mean-centered and scaled to unit variance, and the response was log10-transformed (MFI). The number of latent variables was selected by cross-validation using the minimum cross-validated RMSE criterion. The latent variable optimization curves are reported in Figure 6. The final PLS predicted-versus-observed plots are reported in Figure 7, and the global PLS performance comparison is reported in Figure 9 and Table 2.

Variable Importance in Projection (VIP) scores were calculated to identify influential predictors within each PLS model. Variables with VIP ≥ 1 were treated as influential within the corresponding architecture-specific model. Because the PLS models were independently calibrated and did not always contain identical predictor sets, VIP magnitudes were interpreted as within-model rankings and architecture-dependent patterns rather than as absolute quantities directly comparable across pools. The VIP results are reported in Figure 11.

To further evaluate model robustness beyond average cross-validation metrics, prediction uncertainty was quantified via repeated five-fold cross-validation with independently randomized data partitions. The resulting distributions of cross-validated R^2^, RMSE, and MAE are summarized in Supplementary Figure S1, whereas the corresponding statistical descriptors are reported in Supplementary Table S5. The relatively narrow confidence intervals observed across the industrial datasets demonstrate that the ranking of the predictive models remains stable under different resampling conditions, indicating that the reported performance is not an artifact of a particular data partition. These results provide additional evidence supporting the statistical robustness and reproducibility of the proposed architecture-aware modeling framework.

### 2.9. Supervised Machine Learning Models

To evaluate whether nonlinear models could improve MFI prediction beyond the PLS baseline, five supervised modeling approaches were compared: PLS, PCR-Ridge, Random Forest, Extra Trees, and Gradient Boosting. This set of models was selected to cover linear latent variable regression, regularized linear prediction, and nonlinear tree-based ensemble learning. The model comparison is reported in Figure 10, and the best-performing model for each dataset is summarized in Table 3.

PCR-Ridge was implemented by first projecting the scaled process variables onto principal components and then fitting a ridge regression model to the retained component scores. Ridge regression was included because it provides a regularized linear baseline for collinear industrial datasets [28]. The number of retained principal components and the ridge regularization strength were selected by cross-validation.

Random Forest regression was implemented as an ensemble of decision trees trained on bootstrap samples of the training data and random subsets of predictors [29,30]. This model was included because it can capture nonlinear interactions among process variables while remaining relatively robust to collinearity and outliers.

Extra Trees regression was implemented as an ensemble of extremely randomized trees [31]. In contrast to Random Forest, Extra Trees introduces additional randomness in the split thresholds, which can reduce variance and improve generalization in small-to-moderate industrial datasets. Extra Trees was particularly relevant for the impact and R+I pools, where the process–MFI relationship was expected to be nonlinear and architecture-dependent.

Gradient Boosting regression was implemented as an additive ensemble of sequentially fitted weak learners [32]. This model was included because it can iteratively correct prediction errors and is well suited to heterogeneous datasets in which different regions of the feature space exhibit distinct local relationships.

**Figure 14:**
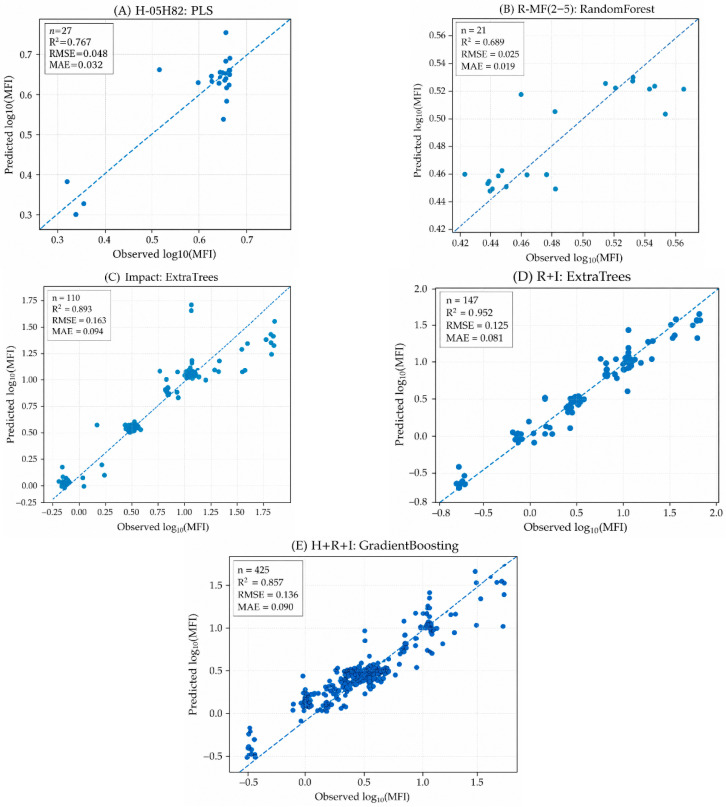
Predicted-versus-observed log10(MFI) values obtained using the best-performing model for each industrial polypropylene dataset. Panels correspond to (**A**) H-05H82 using PLS, (**B**) R-MF(2–5) using Random Forest, (**C**) impact copolymer using Extra Trees, (**D**) random + impact copolymers using Extra Trees, and (**E**) the full polypropylene portfolio using Gradient Boosting. The dashed diagonal represents the 1:1 reference line.

**Figure 15:**
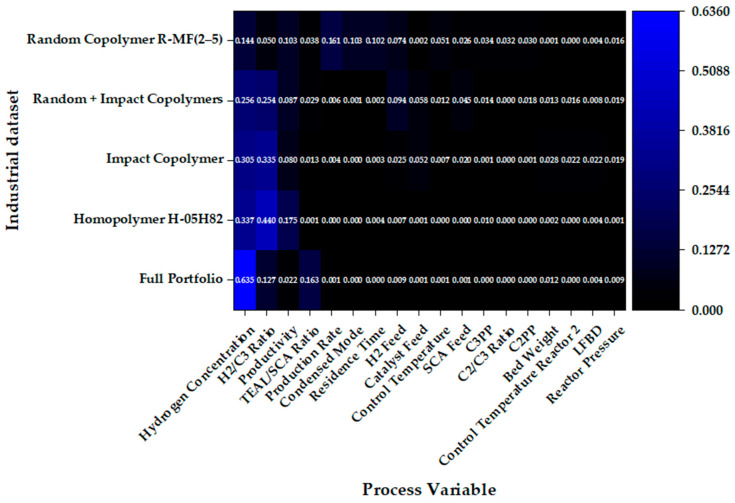
Feature importance obtained from tree-based machine learning models across the industrial polypropylene datasets. Importance values quantify the relative contribution of each process variable to the prediction of log10(MFI) within each dataset. For H-05H82, the best available tree-based model was used for feature importance analysis because the best predictive model was PLS.

All machine learning models were implemented using scikit-learn [29]. Hyperparameters were optimized independently within each modeling pool using the same cross-validated search strategy. The evaluated search ranges are reported in Supplementary Table S3, whereas the selected hyperparameters are summarized in Supplementary Table S6. The same scoring criterion and validation philosophy were applied to Random Forest, Extra Trees, and Gradient Boosting to avoid preferential tuning of any individual algorithm. The best-performing model for each pool was subsequently used to generate the predicted-versus-observed plots in Figure 14 and the feature importance analysis in Figure 15.

### 2.10. Feature Importance Analysis

Tree-based feature importance was calculated for the best available tree-based model in each dataset. For datasets where the best predictive model was already tree-based, the feature importance was extracted from that model. For H-05H82, where PLS remained the best-performing model, the best available tree-based model was used only for feature importance comparison. This choice allowed the same tree-based interpretability framework to be applied across all datasets.

Tree-based feature importance values were normalized within each fitted model. Accordingly, the heatmap in Figure 15 is used to compare the qualitative ranking patterns and the recurrence of dominant descriptors, not absolute importance magnitudes across independently trained models. The analysis complements, rather than replaces, PLS VIP interpretation and the mechanistic evidence from polymerization kinetics.

### 2.11. Model Validation

Three validation protocols were applied.

Protocol A was within-pool five-fold cross-validation. Each modeling pool was divided into five folds. Each model was trained on four folds and validated on the remaining fold. The procedure was repeated until each fold had served as validation data. The resulting CV-R^2^, RMSE, and MAE values were used to evaluate the predictive performance within the architecture. The PLS results from this protocol are reported in Table 2 and Figure 9, whereas the machine learning benchmarking results are reported in Figure 10 and Table 3.

Protocol B was repeated using five-fold cross-validation. The five-fold procedure was repeated using different random partitions to evaluate the stability of the estimated metrics. The repeated validation results were used to verify that a single favorable split did not dominate the model rankings. Detailed repeated validation statistics are reported in the Supplementary Material.

Protocol C was product group cross-validation. This protocol was used to evaluate cross-family transferability. Instead of randomly mixing observations from all grades, validation was organized according to product or architecture groups. This strategy tests whether a model trained within one portion of the industrial portfolio can generalize to another product group. The transferability results are reported in Figure 13 and Table 4.

The same preprocessing and scaling logic was applied consistently within each validation protocol. Scaling parameters were estimated from the training fold and applied to the corresponding validation fold to avoid information leakage. The reported metrics were CV-R^2^, RMSE of log10(MFI), and MAE of log10(MFI).

### 2.12. Statistical and Computational Implementation

All data processing, chemometric modeling, and machine learning analyses were performed using Python version 3.14.6 and scikit-learn version 1.9.0. PCA, PCR-Ridge, Random Forest, Extra Trees, Gradient Boosting, cross-validation, and model evaluation were implemented using scikit-learn [29]. PLS regression was implemented using the SIMPLS framework and verified against standard chemometric formulations [26,27]. Figures were generated from the processed modeling outputs and organized according to the final figure sequence of the manuscript: Figure 1 for dataset structure, Figure 2 for correlation analysis, Figure 3, Figure 4 and Figure 5 for PCA, Figure 6, Figure 7, Figure 8 and Figure 9 for PLS, Figure 10, Figure 11 and Figure 12 for machine learning benchmarking and interpretation, Figure 13 for transferability, and Figure 14 for the mechanistic framework.

The final statistical interpretation was based on the combined evidence from unsupervised latent space analysis, linear latent variable regression, nonlinear model benchmarking, feature importance analysis, and product group validation. This combined strategy was designed to distinguish between prediction accuracy and physicochemical transferability across polypropylene architectures.

## 3. Results and Discussion

### 3.1. Statistical Structure of the Industrial Polypropylene Portfolio

The industrial portfolio was first examined to determine whether the MFI response could be treated as a single homogeneous quality variable or whether it was intrinsically segmented by the polypropylene architecture. This initial step is essential because the three families included in the portfolio (homopolymers, random copolymers, and impact copolymers) do not differ only in commercial classification. They differ in the molecular origin of melt flow, the role of comonomer incorporation, the presence or absence of a rubber phase, and the process variables that dominate their final rheological response [3,4,5,6,7]. Therefore, the statistical structure of the MFI must be interpreted as a first approximation to the underlying architecture–process–property relationship.

The statistical analyses presented in the following sections should therefore be interpreted within this physicochemical framework. Although all production grades are evaluated through the same industrial quality variable (MFI), the molecular origin of melt flow differs substantially among polypropylene architectures. Homopolymers are primarily governed by molecular weight control; random copolymers combine molecular weight and comonomer effects, whereas impact copolymers introduce additional contributions arising from heterophasic morphology. Consequently, similar process variables may acquire different statistical significance depending on the polymer architecture represented in each dataset.

The distribution of the MFI reveals a clear architecture-dependent organization of the industrial portfolio. Homopolymer grades are concentrated within a comparatively narrow MFI range, whereas impact copolymers display the broadest dispersion and dominate the high-flow region of the dataset. Random copolymers occupy an intermediate domain, but their distribution is less populated and more skewed because they represent a smaller fraction of the production runs and commercial grades. The portfolio is therefore not a single unimodal rheological population; it is a multigrade industrial dataset in which each polypropylene architecture contributes a different section of the MFI landscape.

This observation is not merely statistical. It reflects the different molecular origins of the MFI in each architecture. In homopolymers, melt flow is primarily governed by hydrogen-mediated chain transfer, which regulates the average molecular weight and, therefore, the apparent flow response under standardized MFI conditions [8,11,24]. In random copolymers, the response is no longer determined only by the chain length. Ethylene insertion interrupts isotactic propylene sequences, reduces lamellar regularity, modifies crystallinity, and introduces a comonomer content axis that acts together with molecular weight control [3,5,6]. In impact copolymers, the MFI response becomes even more structurally complex because the final material contains a semicrystalline polypropylene matrix and a dispersed ethylene–propylene rubber phase. The rubber-phase fraction, particle morphology, interphase structure, and matrix molecular weight jointly influence the apparent flow behavior [4,7,17].

Thus, Figure 1 establishes the first central result of the study: the industrial MFI space is architecture-stratified before any chemometric or machine learning model is applied. This has direct modeling consequences. If a model is trained on the entire portfolio without explicitly considering the architecture, it must learn a single mathematical relationship across materials whose MFI values arise from different physicochemical mechanisms. This explains why the subsequent PCA, PLS, and ML analyses cannot be interpreted simply as statistical exercises; they are diagnostic tools for evaluating whether polypropylene architectures can be collapsed into a universal prediction domain [9,15,19,20,21,22,23].

After establishing the architecture-dependent distribution of the MFI, the correlation structure among process variables was analyzed. Pearson correlation analysis provides the first linear description of how molecular weight control variables, catalyst-related descriptors, reactor conditions, and hydrodynamic variables are associated with the MFI and with one another.

Hydrogen-related descriptors dominate the correlation matrix. The hydrogen concentration, H2/C3 ratio, and H2 feed show the strongest associations with the MFI, confirming that the principal operational route for controlling melt flow remains chain transfer regulation. This behavior is consistent with the open-loop polymerization mechanism: increasing hydrogen availability promotes chain transfer, reduces the average molecular weight, and increases the MFI [8,11,24].

However, the correlation map also shows that the MFI is embedded in a broader multivariable process network. The catalyst feed, TEAL flow, TEAL/SCA ratio, SCA feed, productivity, bed weight, reactor pressure, residence time, condensed mode operation, and fluidization descriptors form correlated blocks. These blocks reflect the coupled nature of industrial gas-phase polymerization: molecular weight control is linked to active site concentration, donor selectivity, monomer and comonomer partial pressures, heat removal, residence time distribution, and bed hydrodynamics [8,15,24]. Therefore, hydrogen is the dominant molecular weight descriptor, but it is not sufficient to explain the full industrial MFI response.

This point is important because Pearson correlation only captures linear pairwise relationships. It cannot distinguish whether a variable contributes directly to the MFI, serves as a surrogate for grade architecture, or is collinear with another manipulated variable under closed-loop control. For example, industrial feedback trim can invert or attenuate raw hydrogen–MFI relationships because hydrogen is adjusted in response to laboratory MFI deviations [8,11,15]. Consequently, the statistical role of hydrogen in plant data is not identical to its open-loop kinetic role. Figure 4, Figure 8 and Figure 12 therefore extend the correlation analysis by identifying how variable importance changes across architectures and modeling frameworks.

From a chemometric perspective, these physicochemical differences imply that each polypropylene architecture occupies a distinct multidimensional process–property space. If the dominant mechanisms controlling the MFI differ among architectures, one should expect differences in covariance structure, latent dimensionality, variable importance, and ultimately predictive model transferability. Principal component analysis, therefore, provides an appropriate first tool for evaluating whether these industrial datasets can be represented within a common latent domain or whether the architecture itself defines statistically independent process spaces.

Because each polymer architecture was initially analyzed with an independent PCA model, an additional global principal component analysis was performed on the complete industrial polypropylene portfolio to assess whether the observed architectural domains persist in a unified latent space. As shown in Supplementary Figure S2, the three polypropylene architectures remain organized into distinguishable latent regions despite being projected onto a common chemometric space. This observation indicates that the latent domain separation is not an artifact of independent PCA calibration but rather reflects intrinsic physicochemical differences associated with the polymer architecture, reactor configuration, and the evolution of the molecular structure.

### 3.2. Architecture-Dependent Latent Domains Revealed by PCA

The capacity of each industrial dataset to be represented in a reduced latent space was evaluated using the variance explained by the first two principal components. This analysis tests whether each architecture can be compressed into a small number of latent directions or whether architectural complexity increases the dimensionality of the process–property space. PCA was used here as an exploratory chemometric tool to diagnose the multivariate structure, variable correlation, and latent domain organization [9,25].

The cumulative variance explained by PC1 and PC2 changes substantially across datasets. The random copolymer dataset shows the highest PC1+PC2 compression, whereas the impact copolymer dataset shows the lowest cumulative variance. The full portfolio, despite containing the largest number of observations, also shows limited two-component compression. This behavior demonstrates that the number of production runs is not the only factor controlling the PCA structure. Instead, the relevant factor is the number of independent physicochemical mechanisms represented within each dataset.

The random copolymer dataset behaves as the most compressible domain because its MFI is governed by a relatively compact combination of two axes: hydrogen-mediated molecular weight control and ethylene-mediated comonomer incorporation. In this family, ethylene insertion reduces crystallinity and modifies chain regularity, but the material remains monophasic and is produced under a single-reactor logic [3,5,6]. This explains why a small number of latent variables can capture a high fraction of the process variation. The compact latent behavior of the random copolymer pool should be interpreted with appropriate caution because this subset contains fewer production runs and spans a narrower MFI window than the other pools. However, its high compressibility is not treated in this work as evidence of universal simplicity, but as evidence that the evaluated random copolymer grades occupy a restricted and internally coherent industrial domain. The repeated validation results reported in Supplementary Figure S1 and Table S5 show that the predictive behavior of this pool remains stable under resampling, supporting the conclusion that the observed compactness is not driven exclusively by a single favorable data partition.

In contrast, the impact copolymer dataset introduces a heterophasic structure and second reactor descriptors. Impact copolymers contain a polypropylene matrix produced under homopolymer-like conditions and an ethylene–propylene rubber phase generated under a separate gas composition and residence time regime [4,7,17]. The rubber phase introduces independent descriptors, including the EPR fraction, rubber molecular weight, particle morphology, and interfacial dispersion. These features are not simple extensions of the molecular weight axis; they represent additional structure–property dimensions. Therefore, the lower PC1+PC2 variance in the impact dataset is not a statistical artifact but a reflection of higher physicochemical dimensionality. The reduced PC1+PC2 compressibility of the impact copolymer pool should not be attributed only to its larger sample size. If sample size were the dominant factor, the full portfolio would be expected to behave simply as a smoother version of the individual domains. Instead, the impact pool shows reduced compression because it contains additional independent physicochemical axes absent from homopolymer and random copolymer systems. These include matrix molecular weight regulation, second reactor gas composition, rubber-phase formation, bed behavior, residence time effects, and phase morphology. Therefore, the broader latent domain reflects the coexistence of multiple structure–property pathways within a coherent heterophasic architecture, rather than merely routine process noise.

The full portfolio combines homopolymer, random copolymer, and impact copolymer architectures. Its lower compression relative to architecture-restricted domains indicates that a universal latent space must average several distinct pathways: molecular weight control in homopolymers, comonomer-driven crystallinity modification in random copolymers, and rubber-phase morphology in impact copolymers [3,4,5,6,7]. This provides the first quantitative evidence that the architecture is a non-collapsible factor in industrial MFI modeling.

The PCA score plots were used to evaluate whether the production runs occupy common or architecture-specific regions in the PC1–PC2 space. This step translates the numerical summary of variance into a geometric representation of the industrial process domains.

The PCA score distributions reveal architecture-specific latent domains. The homopolymer dataset forms a comparatively compact region, consistent with the simpler molecular origin of the MFI in isotactic polypropylene in the absence of comonomer feed. The random copolymer dataset occupies a distinct score region in which the combined action of hydrogen and ethylene incorporation governs variation. The impact copolymer dataset spans a broader, more dispersed domain, reflecting the additional degrees of freedom associated with heterophasic morphology and second reactor operation [3,4,5,6,7,17].

When random and impact copolymers are combined, the score space becomes more heterogeneous. This heterogeneity increases further in the full portfolio, where homopolymers, random copolymers, and impact copolymers are represented simultaneously. The score space geometry therefore shows that the portfolio is not simply a larger dataset; it is a union of partially incompatible latent domains. This observation is consistent with the study’s original premise: MFI prediction across polypropylene families is limited by architecture-dependent process–structure–property relationships.

The significance of this result extends beyond PCA visualization. In industrial soft sensors, the assumption behind a universal model is that all observations can be represented in a common latent or feature space [8,9,10,15]. Figure 3 challenges that assumption. If each architecture occupies a different region of the score space, then a global model must either learn domain-specific subrelationships or average them into a less interpretable regression. This explains why architecture-restricted PLS models can perform acceptably, whereas universal PLS calibration deteriorates. It also explains why nonlinear ensemble models later improve performance: they can partition the score or feature space into local regions that partially correspond to the material architecture [16,17,18,30,31,32].

Thus, Figure 3 provides the geometric basis for the rest of the study. Figure 4 identifies which process variables define these domains; Figure 7 tests whether a linear latent variable model can estimate the reactor-grade MFI for real-time process monitoring within them; Figure 10 evaluates whether nonlinear models can recover performance; and Figure 13 determines whether the learned relationships are transferable across product families.

To identify the operational variables responsible for the PCA score space structure, loading plots were analyzed for each dataset. These plots determine which process descriptors orient PC1 and PC2 and whether the same variables play equivalent roles across architectures.

The loading plots show that the orientation of the latent variables changes across datasets. In the homopolymer domain, the dominant directions are associated with the hydrogen concentration, H2/C3 ratio, productivity, catalyst feed, and thermal or hydrodynamic variables. This is consistent with a single-reactor iPP process in which the MFI is primarily controlled by the molecular weight but is also modulated by the catalyst productivity, donor selectivity, residence time, and heat transfer stability [8,11,24].

In the random copolymer domain, the loading structure shifts toward a combined molecular weight/comonomer axis. Hydrogen descriptors remain important, but variables associated with ethylene incorporation and C2/C3 balance gain interpretive relevance. This shift is chemically meaningful: in random copolymers, ethylene modifies the crystallization behavior, lamellar regularity, melting temperature, and chain mobility [3,5,6]. Therefore, the statistical loading structure reflects the dual origin of the random copolymer MFI: molecular weight and comonomer-induced microstructural disruption.

In the impact copolymer domain, the loading structure becomes more distributed. Variables related to productivity, reactor pressure, bed inventory, fluidization behavior, feed rates, and second reactor descriptors contribute more strongly. This is expected because impact copolymers are produced through a two-stage architecture: matrix formation and rubber-phase formation [4,7,17]. The MFI in this domain is influenced not only by the matrix molecular weight but also by the EPR phase, phase morphology, and process conditions that determine rubber incorporation.

The key conclusion from Figure 4 is that the same process variable does not have a universal meaning across polypropylene architectures. Hydrogen is always chemically relevant, but its statistical loading depends on the architecture and on the feedback-controlled operating context. Catalyst and donor variables may be secondary in one domain and important in another. Second reactor descriptors are irrelevant to homopolymer and random copolymer production but become essential for impact copolymers. This loading rotation is the chemometric expression of architectural non-collapsibility [9,15,25]. It explains why a global PLS model becomes lossy: it must compress several different process–property maps into a single set of latent directions. The reduction in latent space compression was summarized by comparing the variance captured by PC1 and PC2 across the industrial datasets. The term “loading rotation” is used here in a comparative chemometric sense to describe the architecture-dependent reorientation of the dominant process-variable contributions in PC1–PC2 space. A formal Procrustes or subspace angle metric was not used as the primary criterion because the predictor sets are not identical across all architecture pools and some descriptors are architecture-specific. Under these conditions, direct geometric alignment of loading vectors may obscure rather than clarify the physicochemical interpretation. Therefore, the conclusion is based on the combined evidence of PCA loading redistribution, architecture-specific VIP patterns, tree-based feature importance changes, and product group validation, rather than on visual inspection alone.

The PC1+PC2 variance decreases when the dataset moves from architecture-restricted domains to impact-rich and full-portfolio domains. This result confirms that architectural complexity increases the number of relevant latent dimensions. The effect is not simply numerical; it has a physical origin. Homopolymers are monophasic and are mainly controlled by molecular weight variation. Random copolymers add ethylene incorporation but retain a single-phase architecture. Impact copolymers add a second phase and second reactor operation [3,4,5,6,7,17]. The full portfolio combines all these mechanisms.

The decrease in explained variance means that a two-dimensional latent description becomes progressively less adequate as the portfolio includes more structural mechanisms. This is important for model selection. PLS relies on the assumption that a small number of latent variables can capture most of the relevant covariance between process variables and the response [10,26,27]. When PCA already shows reduced compression, the subsequent PLS model is expected to require either more latent variables or to lose predictive efficiency. This is exactly what is observed in the PLS block.

Figure 5 also explains why nonlinear models become attractive. Tree-based algorithms do not require the entire dataset to be represented by a single global latent direction. Instead, they can split the feature space into local regions and approximate separate relationships within each region [30,31,32]. Therefore, the loss of PCA compressibility is not merely descriptive; it is a mechanistic predictor of why linear PLS models weaken and why ensemble ML methods improve performance in heterogeneous polypropylene portfolios.

### 3.3. PLS Modeling of MFI

PLS models were developed to establish a linear latent variable baseline for MFI prediction. Before evaluating predictive performance, the number of latent variables was optimized via cross-validation to avoid overfitting and determine the amount of latent complexity required for each architecture. PLS was selected because it is a standard chemometric approach for collinear industrial data and soft sensor development [10,26,27].

The optimal number of latent variables is strongly architecture-dependent. The random copolymer dataset reaches its minimum prediction error with a single latent variable, whereas the impact and R+I datasets require more components. The homopolymer dataset requires an intermediate number of latent variables, and the full portfolio reaches its optimum with fewer components than the impact-rich datasets but with poorer global predictive quality.

The single-latent-variable behavior of the random copolymer dataset is mechanistically meaningful. Random copolymers combine two dominant effects: hydrogen-mediated molecular weight control and ethylene-mediated modification of chain regularity [3,5,6,8]. Within the evaluated random copolymer pool, these effects appear sufficiently coupled to be represented by one main latent direction. This supports the interpretation that random copolymers form a compact latent domain, as suggested by Table 1 and Figure 5.

The impact and R+I datasets require more latent variables because their MFI response is not controlled by one dominant axis. Impact copolymers contain a matrix phase and a rubber phase, and the final melt flow response depends on the matrix molecular weight, EPR fraction, phase morphology, second reactor conditions, and hydrodynamic variables [4,7,17]. When random and impact copolymers are combined, the model must simultaneously account for comonomer-driven chain irregularity and rubber-phase morphology. This increased latent complexity explains the higher number of components required for stable PLS prediction.

The full portfolio presents a different problem. Although the optimum is reached with fewer components than in impact-rich datasets, this does not mean that the portfolio is simpler. It means that the global PLS model compresses several architecture-specific relationships into a limited number of compromise directions. This compression is lossy: it reduces model complexity but sacrifices architecture-specific interpretability and transferability. This behavior is central to the paper’s argument that universal linear calibration is limited by physicochemical heterogeneity [15,19,20,21,22,23].

After latent variable optimization, the predictive behavior of each PLS model was evaluated by comparing the cross-validated predicted and observed log10(MFI) values.

The PLS predicted-versus-observed plots confirm that linear latent variable modeling is effective within restricted architecture domains but less effective when multiple architectures are combined. The homopolymer and random copolymer datasets remain relatively close to the 1:1 reference line. In contrast, the impact, R+I, and full-portfolio datasets show broader dispersion, indicating that the same latent variable strategy becomes less precise as the process–structure–property space becomes more heterogeneous.

For homopolymers, this behavior is expected because the MFI is mainly associated with the molecular weight and hydrogen-mediated chain transfer [8,11,24]. Although the catalyst productivity, donor balance, reactor pressure, and fluidization conditions modulate the final response, the molecular origin of the MFI remains comparatively compact. For random copolymers, the prediction remains acceptable because the comonomer axis is still integrated within a single-reactor process [3,5,6]. The model can therefore represent the combined effects of hydrogen and ethylene incorporation without requiring a strongly nonlinear partitioning of the data.

For the impact and mixed datasets, the broader prediction dispersion reflects the inability of a single PLS space to capture heterophasic morphology and architecture-specific operating regimes. The impact copolymer MFI is not determined only by the matrix molecular weight. It is also affected by the rubber phase, the second gas composition profile, residence time, bed behavior, and productivity in the second reactor [4,7,17]. In the R+I and full-portfolio datasets, these effects coexist with random copolymer comonomer effects and homopolymer molecular weight control. Therefore, the model is not simply less accurate; it is forced to average non-equivalent mechanisms.

This distinction is important for industrial deployment. A PLS soft sensor may perform well when calibrated and used within a single architecture, but its reliability decreases when it is applied as a universal model across grade families. This limitation aligns with the multigrade soft sensor literature, where transfer learning, domain adaptation, or local model strategies are required to recover predictive performance across operating regimes [8,15,19,20,21,22,23].

The PLS models were further interpreted using the Variable Importance in Projection scores. VIP analysis identifies which process descriptors most strongly support the prediction of log10(MFI) within each architecture.

The VIP heatmap confirms that hydrogen-related descriptors are the most persistent predictors of the MFI. The hydrogen concentration, H2/C3 ratio, and H2 feed consistently exhibit high VIP values, supporting the central role of chain transfer in MFI regulation [8,11,24]. This result agrees with the Pearson correlation structure in Figure 2 and with the mechanistic role of hydrogen in gas-phase polypropylene production.

Nevertheless, the VIP structure is not identical across architectures. In homopolymers, the dominant VIP descriptors remain close to the molecular weight control axis, together with catalyst and productivity variables that modulate the polymerization rate and active site behavior. In random copolymers, variables related to comonomer incorporation and C2/C3 composition gain relevance because both the chain length and ethylene-induced changes influence the MFI in chain regularity and crystallinity [3,5,6]. In impact copolymers, the reactor pressure, bed variables, catalyst feed, SCA feed, production rate, and second reactor descriptors become more relevant, reflecting the additional contribution of heterophasic morphology and process hydrodynamics [4,7,17].

Therefore, the VIP analysis does not simply rank variables; it reveals how the mechanism of MFI prediction changes across architectures. Hydrogen remains the shared molecular weight descriptor, but the secondary descriptors are architecture-specific. This result directly supports the PCA loading rotations observed in Figure 4. It also provides a bridge to the tree-based feature importance analysis in Figure 12, where nonlinear models retain hydrogen as a dominant descriptor but assign additional importance to variables involved in catalyst behavior, reactor operation, and second reactor morphology development.

The numerical performance of the optimized PLS models is summarized in Table 2.

The PLS metrics show that model performance is controlled more by the architecture than by sample size. Architecture-restricted models reach satisfactory CV-R^2^ values, whereas the full portfolio presents the lowest PLS performance despite containing the largest number of observations. This result demonstrates that adding more production runs does not necessarily improve a linear latent variable model if those runs introduce new physicochemical mechanisms.

The high performance in the impact dataset, despite its structural complexity, indicates that PLS can still be effective when the data belong to a coherent architecture domain. Within impact copolymers, the process variables and morphology-related descriptors remain internally consistent [4,7,17]. The model does not need to transfer across fundamentally different material families; it only needs to represent the variation inside the impact domain. In contrast, the full portfolio requires the model to describe the behavior of homopolymers, random copolymers, and impact copolymers simultaneously. This produces a lower global CV-R^2^ because a single latent space cannot preserve all architecture-specific directions.

This distinction is essential. The issue is not that PLS is intrinsically weak. Rather, PLS becomes limited when the covariance structure is not shared across architectures [10,26,27]. When the latent directions are stable, PLS provides a defensible baseline. When the latent directions rotate across domains, as shown in Figure 4, the global model becomes a compromise. Table 2, therefore, provides the quantitative basis for evaluating nonlinear ML methods: the goal is not merely to improve R2, but to determine whether nonlinear models can compensate for architecture-dependent non-collapsibility.

The PLS performance metrics were compared visually to evaluate how the predictive accuracy and prediction error evolve across polypropylene domains.

The PLS performance comparison confirms the architecture dependence observed in the predicted-versus-observed plots. The lowest errors occur in the homopolymer and random copolymer datasets, whereas the R+I dataset shows the largest prediction error. The full portfolio exhibits reduced explained variance compared with architecture-specific models, indicating that a universal PLS calibration cannot fully resolve the mixed-architecture space.

The R+I result is especially informative. This dataset combines random copolymers, whose MFI depends on molecular weight and comonomer incorporation, with impact copolymers, whose response also depends on rubber-phase morphology and second reactor operation [3,4,5,6,7,17]. The resulting dataset is not simply larger; it is structurally mixed. The increased RMSE and MAE therefore reflect a loss of mechanistic coherence. The model must represent two distinct architecture-dependent pathways using one linear latent space.

Figure 9 supports the transition toward nonlinear ML models. If the prediction error was caused by insufficient sample size or noise, nonlinear models would not necessarily offer a systematic advantage. However, if the error arises from nonlinear and domain-dependent interactions, tree-based models should improve performance by partitioning the feature space [30,31,32]. This hypothesis is tested directly in Figure 10.

### 3.4. Machine Learning Benchmarking

To determine whether nonlinear algorithms could better capture architecture-dependent relationships, PLS and PCR-Ridge were benchmarked against Random Forest, Extra Trees, and Gradient Boosting using the same industrial domains. The model family was selected to include linear latent variable modeling, regularized linear regression, and nonlinear ensemble learning [26,27,28,29,30,31,32]. Importantly, the machine learning models used in this work are not interpreted as replacements for polymerization kinetics or polymer physics. Their role is restricted to identifying nonlinear statistical relationships within industrial process data. The physicochemical interpretation is instead derived from the known Ziegler–Natta polymerization mechanisms, the reactor configuration, the architecture-specific process descriptors, PCA loading behavior, PLS VIP scores, and tree-based feature importance. Therefore, the models are not used as black box evidence of causality. Rather, they serve as diagnostic tools to reveal whether the same reactor variables preserve equivalent predictive meaning across homopolymer, random copolymer, and impact copolymer domains. This distinction is essential because the central conclusion of the study is not that machine learning can universally estimate the reactor-grade MFI for real-time process monitoring, but that the polymer architecture imposes physicochemical constraints on the transferability of any data-driven MFI model.

The benchmarking heatmap shows that tree-based ensemble models outperform linear approaches in the most heterogeneous datasets. Extra Trees provides the best performance in the impact and R+I domains, while Gradient Boosting provides the best result for the full portfolio. In contrast, PLS remains competitive in the homopolymer dataset, where the process–MFI relationship is closer to a compact latent variable structure.

This pattern is consistent with the physicochemical interpretation developed from PCA and PLS. Tree-based models can split the process space into local regions, which is advantageous when different architectures occupy different latent domains [30,31,32]. Instead of forcing all samples into a single global projection, ensemble models can approximate separate relationships for different ranges of hydrogen concentration, catalyst feed, TEAL/SCA ratio, productivity, reactor pressure, comonomer content, and second reactor variables. This explains why the improvement is strongest in the architecturally heterogeneous datasets.

However, the benchmarking result must not be overinterpreted as proof that ML eliminates the architecture problem. The improved CV-R^2^ values show that nonlinear algorithms fit the within-pool structure better than PLS. They do not prove that the resulting models transfer well across product families. In industrial deployment, this distinction is critical. A model can predict well inside a mixed historical dataset while still failing when applied to a new grade or an underrepresented architecture. Therefore, Figure 10 demonstrates algorithmic gain, but Figure 13 is required to evaluate transferability [15,19,20,21,22,23].

The benchmarking also supports an operational recommendation. For single-family homopolymer monitoring, a linear latent variable model may be sufficient and more interpretable. For impact-rich or full-portfolio monitoring, tree-based models are preferable because they capture nonlinear interactions. Nevertheless, their implementation should be coupled with architecture labels, product group validation, or domain adaptation strategies to prevent misleading universal deployment [16,17,18,19,20,21,22,23].

The best-performing model from each dataset was evaluated using predicted-versus-observed log10(MFI) values to verify whether the higher CV-R^2^ values correspond to improved pointwise prediction.

The best-performing models improve the agreement between the predicted and observed log10(MFI), particularly in the impact, R+I, and full-portfolio datasets. Extra Trees provides strong predictions for the R+I domain, while Gradient Boosting improves full-portfolio predictions relative to the PLS baseline. The predicted values are more tightly distributed around the 1:1 line than in the linear PLS models, confirming that nonlinear algorithms better capture the internal structure of heterogeneous datasets.

The improvement has a clear mechanistic interpretation. In impact copolymers, the relationship between the reactor variables and MFI is not globally linear because the final response depends on the matrix molecular weight, the rubber-phase contribution, and the second reactor conditions [4,7,17]. Extra Trees can approximate this behavior by building multiple decision partitions [31]. In the full portfolio, Gradient Boosting improves prediction by sequentially correcting errors across different regions of the architecture space [32]. This makes it suitable for a dataset in which homopolymer, random copolymer, and impact copolymer domains coexist.

However, the full-portfolio panel still shows residual dispersion, especially where the MFI values span wider rheological ranges. This means that nonlinear learning improves predictive accuracy but does not erase the underlying physicochemical heterogeneity. The model can learn local patterns, but it still operates on a dataset whose samples come from different material architectures. Therefore, Figure 11 should be interpreted together with Figure 13. High within-pool prediction demonstrates algorithmic capacity; product group validation tests whether the learned relationship is transferable [15,19,20,21,22,23].

This distinction is one of the study’s main contributions. Many soft sensor studies report improved prediction using advanced algorithms, but fewer evaluate whether the model remains valid across product families [8,15,19,20,21,22,23]. The present results show that predictive accuracy and transferability are related but not equivalent. A high CV-R^2^ within the training domain is necessary but not sufficient for robust deployment across a multigrade polypropylene portfolio.

Feature importance was used to interpret the tree-based models and to identify whether the nonlinear algorithms relied on the same chemical descriptors as PLS or incorporated additional architecture-specific variables.

The hydrogen concentration and the H2/C3 ratio remain dominant in most tree-based models. This confirms that the nonlinear algorithms preserve the primary chemical mechanism already identified by Pearson correlation and PLS VIP analysis: hydrogen-mediated chain transfer is the central driver of molecular weight control and MFI regulation [8,11,24]. Therefore, the ML models do not replace the chemical interpretation of the system; they extend it.

The additional value of feature importance lies in identifying secondary nonlinear contributors. The TEAL/SCA ratio, catalyst feed, SCA feed, H2 feed, production rate, reactor pressure, bed descriptors, and second reactor variables become important depending on the architecture. These variables represent different physical aspects of the polymerization process. The catalyst feed and TEAL/SCA ratio are related to the active site concentration and donor selectivity. The production rate and bed descriptors reflect the process hydrodynamics and heat balance. The reactor pressure and gas composition control the availability of monomers and comonomers. The second reactor descriptors are directly linked to rubber-phase formation in impact copolymers [4,7,17,24].

This pattern provides an interpretable bridge between chemometrics and machine learning. PLS identifies the dominant linear descriptors, whereas tree-based feature importance reveals additional nonlinear or conditional dependencies [26,30,31,32]. For example, a variable may have low global linear importance but become predictive within a specific architecture or operating window. This explains why tree-based models improve performance in heterogeneous datasets: they do not rely on one universal direction of covariance; they identify conditional variable interactions.

Figure 12 therefore reinforces the main conclusion from Figure 4 and Figure 8. The hydrogen axis is universal, but the secondary descriptors are architecture-dependent. This is precisely why the best algorithm changes across datasets and why transferability must be evaluated explicitly.

The best-performing model for each dataset is summarized in Table 3.

The optimal predictive algorithm changes with the polypropylene architecture. PLS is sufficient for the homopolymer dataset; Random Forest performs best for the random copolymer dataset; Extra Trees dominates the impact on the R+I datasets; and Gradient Boosting provides the best performance for the full portfolio. This pattern indicates that no single learning architecture is universally optimal across all polypropylene families. The difference between Extra Trees and Gradient Boosting can be interpreted in terms of the structure of the calibration domain. Extra Trees performed best in the R+I and impact-rich domains because its highly randomized splits reduce variance and efficiently capture multiple local relationships within structurally complex but internally coherent datasets. In contrast, Gradient Boosting performed best on the full H+R+I portfolio because the complete dataset contains several distinct sequential error sources associated with homopolymer, random copolymer, and impact copolymer regions. The boosting strategy is well suited to this situation because it progressively corrects residual errors across heterogeneous regions of the feature space. Therefore, the difference between the two ensemble methods is consistent with the architecture-dependent organization of the data, although statistical sampling effects cannot be completely excluded.

The homopolymer result is chemically reasonable. Because the molecular origin of the MFI is comparatively compact, a linear latent variable model can capture most of the relevant covariance [8,10,24,26]. The random copolymer result suggests that moderate nonlinearities begin to emerge when ethylene is incorporated, but the domain remains sufficiently structured for Random Forest to perform well [3,5,6,30]. The impact and R+I results show that stronger nonlinear partitioning is beneficial when heterophasic morphology and mixed-architecture effects are present in the dataset [4,7,17,31]. The full portfolio requires Gradient Boosting because the model must iteratively reduce errors across several architecture-specific regions [32].

Therefore, Table 3 supports a practical deployment principle: the model should be selected according to the architectural scope of prediction. A single-family soft sensor can prioritize interpretability and use PLS. A multigrade or full-portfolio soft sensor requires a nonlinear model, but it should not be assumed to be universally transferable without product group validation [15,19,20,21,22,23]. This motivates the transferability analysis in the next section.

### 3.5. Cross-Family Transferability of MFI Prediction Models

Predictive performance within a dataset does not guarantee robustness across product families. Therefore, transferability was evaluated by comparing within-pool performance with product group cross-validation performance. This analysis distinguishes algorithmic fitting capacity from physicochemical generalization. The distinction is consistent with the broader soft sensor literature, where model transferability and domain adaptation are recognized as separate problems from ordinary cross-validation accuracy [15,19,20,21,22,23].

**Figure 16:**
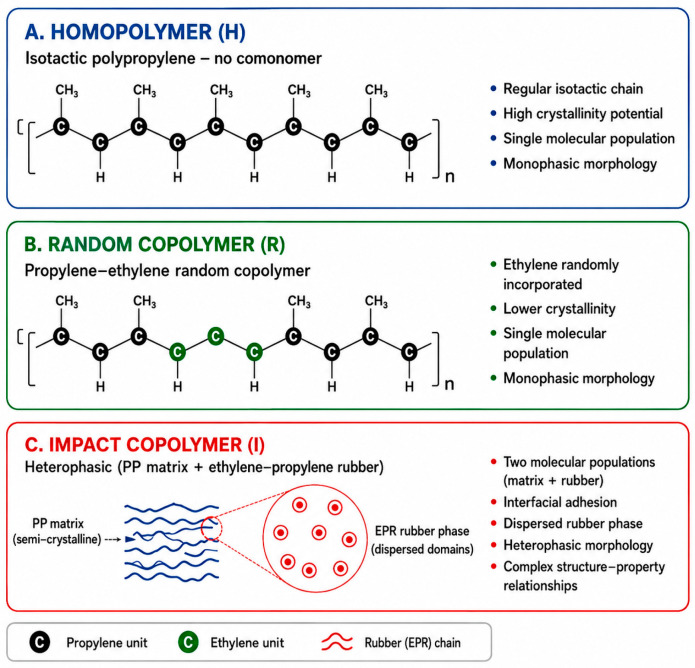
Molecular representation of the three reactor-grade polypropylene architectures.

The molecular distinctions among the three reactor-grade polypropylene architectures underlying the observed transferability behavior are summarized in Figure 16. From an industrial perspective, architecture-dependent transferability also provides a practical criterion for model deployment. Universal predictive models become increasingly unreliable when architecture-specific mechanisms contribute significantly to the final quality response. In practical implementation, architecture-specific calibration should therefore be preferred whenever production portfolios contain heterogeneous molecular structures or multi-reactor products. Universal models remain useful for exploratory monitoring and approximate prediction. In contrast, architecture-aware models are more appropriate for real-time quality control, soft sensor development, digital twins, and advanced process optimization.

The transferability analysis shows that high within-pool performance does not necessarily translate into high cross-family performance. The best ML models achieve strong CV-R^2^ values when trained and validated within the same domain, but performance decreases when validation is organized by product group. This decrease is especially significant in the mixed and full-portfolio datasets, where multiple architecture-dependent relationships coexist.

**Figure 17:**
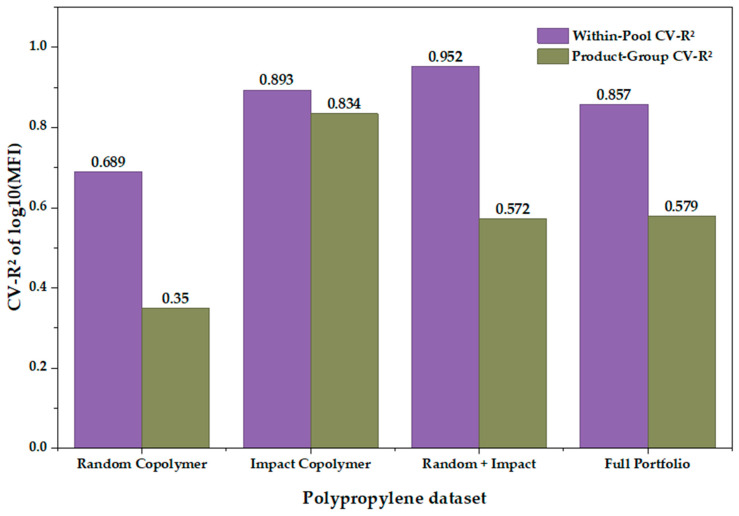
Cross-family transferability of predictive models across polypropylene architectures. The plot compares within-pool PLS performance, within-pool best ML performance, and product group cross-validation performance. The decrease from the best ML performance to the product group CV quantifies the transferability loss associated with applying a model across different polypropylene domains.

As shown in Figure 17, the reduction from within-pool machine learning performance to product group cross-validation performance reveals a substantial loss of predictive transferability across polypropylene architectures. This result is central to the study because it demonstrates that the principal limitation of universal MFI modeling is not simply insufficient algorithmic complexity. Nonlinear models can improve prediction within a heterogeneous dataset, but they cannot fully eliminate the fact that homopolymers, random copolymers, and impact copolymers follow different structure–property pathways [3,4,5,6,7,17]. In other words, model transferability is constrained by polymer architecture. Transferability loss cannot be explained solely by differences in the MFI range. In industrial polypropylene production, homopolymer, random copolymer, and impact copolymer grades may exhibit overlapping MFI values while retaining fundamentally different molecular architectures. Thus, a similar MFI does not imply an equivalent molecular weight distribution, crystallinity, comonomer distribution, rubber-phase content, or melt rheological mechanism. Product group validation therefore evaluates more than a numerical shift in the response variable; it tests whether the learned process–MFI relationship remains valid when the underlying structure–property pathway changes. The observed loss of transferability is consequently interpreted as evidence of a mechanism shift rather than merely a distribution shift.

The R+I domain illustrates this limitation clearly. Within-pool performance is high because the model can learn patterns from both random and impact copolymers. However, product group validation reveals that the learned relationship does not transfer equally well between these families. Random copolymers are governed by molecular weight and comonomer content axes, whereas impact copolymers introduce rubber-phase morphology and second reactor operation [3,4,5,6,7]. These mechanisms are not interchangeable. Therefore, the model can fit the union of both domains but still lose generalization when one product group is withheld.

The full portfolio shows the same phenomenon at a broader scale. The model benefits from the large number of observations and the flexibility of Gradient Boosting, but the product group CV-R^2^ decreases because the model must generalize across the entire H+R+I architecture spectrum. This confirms the architectural non-collapsibility identified by PCA and the variable importance redistribution observed in the PLS and ML models. Figure 13 therefore provides strong evidence that universal calibration is limited by physicochemical transferability, not merely by statistical prediction error [15,19,20,21,22,23].

Table 4 quantifies the transferability loss by comparing the within-pool CV-R^2^ with the product group CV-R^2^.

The numerical transferability metrics confirm the qualitative trends observed in Figure 13. The impact copolymer dataset shows the smallest transferability loss, whereas the R+I dataset shows the largest loss. The full portfolio retains intermediate transferability but still exhibits a substantial decrease relative to its within-pool performance.

The low transferability loss in the impact dataset indicates that, although impact copolymers are structurally complex, they form a coherent architecture domain. Their complexity is internal but consistent: matrix formation, rubber-phase formation, and second reactor descriptors are shared across the family [4,7,17]. The model can therefore generalize more effectively within impact copolymers than across mixed-architecture domains.

In contrast, the high transferability loss in R+I indicates that combining random and impact copolymers creates a domain with conflicting physicochemical origins. Random copolymers and impact copolymers may share ethylene-containing descriptors, but the meaning of ethylene is different. In random copolymers, ethylene is inserted statistically into the polypropylene chain, thereby modifying crystallinity and chain regularity [3,5,6]. In impact copolymers, ethylene is associated with rubber-phase formation and heterophasic morphology [4,7,17]. Therefore, the same descriptor can encode different physical phenomena depending on the architecture. This explains why a model trained on the combined domain can fit well internally but loses transferability under product group validation.

Table 4, therefore, establishes transferability loss as a quantitative descriptor of architectural mismatch. It converts the qualitative concept of different polypropylene families into a measurable modeling penalty. This penalty should be considered in industrial soft sensor deployment, especially during grade changes or when a model trained on one product family is applied to another [15,19,20,21,22,23].

### 3.6. Physicochemical Interpretation of Model Transferability

**Figure 18:**
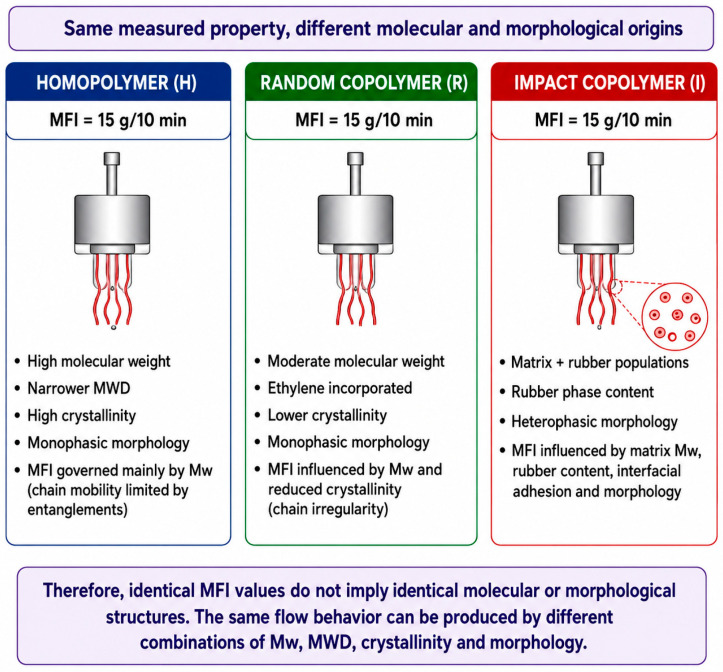
Identical MFI values arising from different physicochemical origins.

Table 5 summarizes the main physicochemical characteristics governing melt flow generation in the three reactor-grade polypropylene architectures. As illustrated in Figure 18, identical MFI values may arise from fundamentally different physicochemical origins depending on the polymer architecture. Building on this comparison, Table 6 integrates the statistical, chemometric, machine learning, and polymer-science results by relating polypropylene architecture to physicochemical complexity, model behavior, and transferability. From a Ziegler–Natta kinetic perspective, the architecture-dependent behavior observed in the models arises from the competition among chain propagation, chain transfer, comonomer insertion, and phase formation. In homopolymers, hydrogen primarily increases the probability of chain transfer events, reducing the average chain length, decreasing the entanglement density, lowering the melt viscosity, and increasing the MFI. In random copolymers, ethylene insertion introduces sequence defects into the polypropylene backbone, reducing stereoregular sequence length, crystallinity, and lamellar perfection; therefore, the MFI reflects both molecular weight control and comonomer-induced modification of chain mobility. In impact copolymers, the second reactor introduces an additional ethylene–propylene rubber phase, so the measured MFI is influenced not only by the matrix molecular weight but also by the rubber content, dispersed-phase morphology, interfacial structure, and matrix–rubber flow coupling. Consequently, the same process descriptor can represent different molecular phenomena depending on the architecture being produced.

The progression from homopolymer to random copolymer and impact copolymer corresponds to a progressive increase in physicochemical complexity. Homopolymers are monophasic isotactic polypropylene materials whose MFI is primarily governed by molecular weight control [8,24]. Random copolymers introduce ethylene units into the polypropylene backbone, reducing the chain regularity, crystallinity, and melting temperature while adding a comonomer content contribution to the flow response [3,5,6]. Impact copolymers introduce the largest structural discontinuity because they combine a semicrystalline polypropylene matrix with a dispersed ethylene–propylene rubber phase [4,7,17].

This architectural hierarchy explains the observed modeling hierarchy. PLS is effective when the response is dominated by a compact latent structure [10,26,27]. Random Forest is useful when moderate nonlinear interactions are present [30]. Extra Trees and Gradient Boosting become more effective when the response depends on multiple conditional relationships among reactor variables, comonomer content, catalyst behavior, and morphology [31,32,33]. Therefore, the model sequence is not arbitrary; it follows the increasing complexity of the material architecture.

Table 5 also explains why transferability does not scale directly with model accuracy. A model may perform very well within an architecture because the underlying mechanism is internally coherent. However, when the model is applied across architectures, the same input variable can correspond to different physical effects. Hydrogen primarily controls the chain length in homopolymers, interacts with comonomer incorporation in random copolymers, and participates in a more complex matrix/rubber-phase balance in impact copolymers [3,4,5,6,7,8,24]. Similarly, the ethylene content is a chain irregularity descriptor in random copolymers but a rubber-phase descriptor in impact copolymers [3,4,5,6,7]. This context dependence is the physicochemical origin of transferability loss.

The practical implication is that the polypropylene architecture should not be treated as metadata after model development. It should be included as a primary modeling descriptor, a stratification variable, or a domain label. Future soft sensors and digital twins for polypropylene production should therefore use architecture-aware modeling strategies rather than assuming that a single universal regression can describe all commercial grades [15,19,20,21,22,23].

### 3.7. Relationship Between Process Variables, PP Architecture, and MFI—Industrial Validation

#### 3.7.1. Mechanistic Framework Linking Process Variables, Polymer Architecture, and MFI

**Figure 19:**
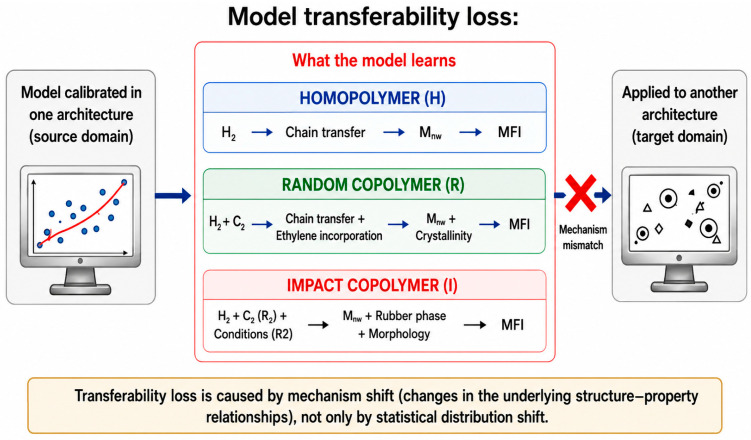
Physicochemical origin of architecture-induced loss of model transferability.

As illustrated in Figure 19, the loss of model transferability arises from architecture-dependent changes in the process–structure–property relationships governing MFI. These relationships are summarized in Table 7. Although the present study analyzes polymer architecture using discrete industrial classes—homopolymer, random copolymer, and impact copolymer—the proposed framework is equally compatible with continuous physicochemical descriptors, such as ethylene incorporation, rubber-phase fraction, crystallinity, molecular weight distribution, and phase volume fraction. These variables represent continuous manifestations of polymer architecture and may provide a more detailed basis for developing future architecture-aware predictive models.

#### 3.7.2. Industrial Validation and Process Control Implications of the Proposed Reactor-Grade MFI Soft Sensor

Unlike conventional laboratory characterization studies, the primary objective of the present work is not to replace polymer science with machine learning, nor to infer polymerization mechanisms directly from statistical models. Instead, the proposed methodology was developed as an industrial soft sensor for reactor-grade polypropylene, in which rapid estimation of the melt flow index (MFI) is essential for real-time process monitoring and operational decision-making. In industrial polypropylene production, the freshly synthesized polymer leaving the reactor is routinely characterized before pelletization, stabilization, additive incorporation, or extrusion. Consequently, the measured MFI reflects the intrinsic outcome of the polymerization process itself rather than the influence of downstream formulation or processing. Under these conditions, the MFI constitutes one of the principal quality indicators used to evaluate whether the reactor is producing the desired molecular architecture and polymer specifications.

Under routine industrial operation, reactor-grade polypropylene samples are periodically collected and transported to the quality control laboratory, where the MFI is determined using a standard melt flow plastometer. Although highly reliable, this analytical procedure requires sample transportation, conditioning, testing, quality verification, and reporting of results before the information becomes available to process engineers. Consequently, laboratory MFI measurements provide retrospective information describing the reactor behavior several tens of minutes after the polymer has already been synthesized. During this interval, deviations in the hydrogen concentration, catalyst productivity, reactor temperature, gas composition, residence time, or comonomer incorporation may continue affecting polymer quality, potentially generating off-specification material, unnecessary energy consumption, reactor fouling, increased raw material losses, and additional reprocessing costs. The proposed architecture-aware model addresses this industrial limitation by estimating the reactor-grade MFI directly from process variables that are continuously measured by the distributed control system (DCS). Rather than replacing laboratory measurements, the proposed soft sensor complements conventional quality control practice by providing reliable MFI estimates before laboratory results become available. This earlier availability of information enables process engineers to anticipate process deviations, optimize reactor operation, minimize off-specification production, and implement corrective actions in near-real time while laboratory confirmation is still pending. Importantly, the predictive capability of the proposed soft sensor should not be interpreted as evidence that machine learning algorithms explain polymerization chemistry. The physicochemical interpretation remains grounded in polymerization kinetics, molecular architecture evolution, process–structure–property relationships, melt rheology, PCA loading behavior, PLS latent variables, and architecture-dependent feature importance. Machine learning provides an efficient computational framework that integrates multiple process variables into a robust predictive model suitable for industrial deployment.

**Figure 20:**
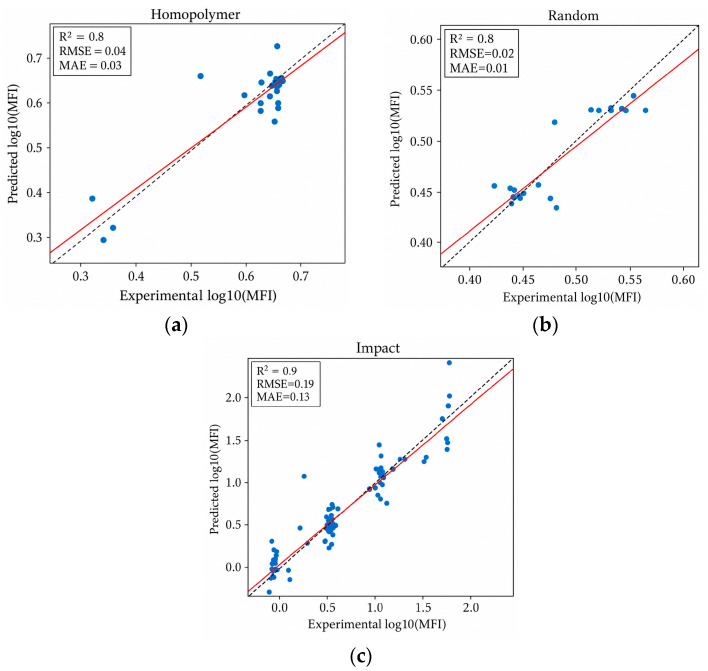
Experimental-versus-predicted reactor-grade melt flow index for the polypropylene architectures. Predicted log10(MFI) values are plotted against the corresponding experimental log10(MFI) values for (**a**) homopolymer, (**b**) random copolymer, and (**c**) impact copolymer datasets.

Figure 20 compares the experimentally measured MFI values obtained using the industrial plastometer with the corresponding values predicted by the proposed architecture-aware soft sensor. The excellent agreement between the two datasets confirms that the predictive models successfully reproduce the laboratory measurements used for routine industrial quality control. Consequently, the proposed methodology provides not only statistically satisfactory performance but also practical engineering information directly applicable to industrial reactor operation.

From an industrial perspective, the value of the proposed methodology is therefore not limited to improving the predictive accuracy. Its principal contribution lies in reducing the time required to obtain actionable quality information. Whereas laboratory measurements characterize reactor performance retrospectively, the proposed soft sensor estimates the reactor-grade MFI in near-real time, enabling earlier process intervention while preserving the physicochemical interpretation provided by polymer science. In this sense, machine learning complements laboratory characterization rather than replacing it.

### 3.8. Scope, Limitations, and Industrial Decision Criteria

Although the proposed architecture-aware modeling framework demonstrated robust predictive performance across multiple industrial polypropylene families, several limitations should be acknowledged. First, the present study was conducted using steady-state production data collected from a single large-scale gas-phase polypropylene plant operating with a single Ziegler–Natta catalyst technology. Consequently, the reported latent structures and transferability boundaries should be interpreted as representative of this industrial system rather than universally applicable across all polypropylene production processes. Second, the transient operating conditions associated with grade transitions were intentionally excluded because they involve continuously changing reactor inventories, residence time distributions, and polymer compositions that violate the steady-state assumptions required for chemometric calibration. Therefore, the proposed models should currently be considered as steady-state soft sensors. Extension toward dynamic grade transition prediction represents an important direction for future research. Because all data were collected from a single industrial gas-phase polypropylene plant using one catalyst technology, the numerical boundaries reported here should not be interpreted as universal constants for all polypropylene processes. Different reactor configurations, catalyst families, hydrogen sensitivities, donor systems, or comonomer incorporation efficiencies may shift the specific model parameters and transferability thresholds. Nevertheless, the central framework proposed in this study is expected to remain generally applicable: whenever different polymer architectures are generated through distinct kinetic, compositional, or morphological pathways, architecture-aware calibration should be preferred over architecture-blind universal modeling. Future work should therefore evaluate the proposed framework using independent plants, alternative catalyst systems, and external production campaigns. An important implication of the present framework is that it remains empirically falsifiable. The proposed architecture-dependent interpretation would require revision if future industrial datasets collected from multiple catalyst technologies, reactor configurations, and production sites demonstrated statistically equivalent latent variable structures, feature importance rankings, and cross-family transferability without requiring architecture-aware calibration. Under such conditions, algorithmic improvements alone would be sufficient to explain universal MFI prediction. The present results do not support that scenario for the industrial portfolio investigated here. The present models were developed for steady-state production and should not be interpreted as dynamic grade transition predictors. During grade transitions, reactor inventories, residence time distributions, gas composition, catalyst productivity, and polymer architecture evolve continuously, producing mixed material populations that do not satisfy the steady-state assumptions used for calibration. For this reason, the proposed architecture-aware framework should be considered a foundation for future dynamic soft sensors rather than a complete transition control model. Its practical extension to grade transitions would require time-resolved data, residence time modeling, moving window calibration, and explicit tracking of the fraction of the architecture that is evolving during the transition.

Third, although the portfolio comprises multiple commercial polymer architectures spanning a broad industrial operating space, external validation through independent production campaigns or across different manufacturing sites was beyond the scope of the present work. Future studies, including additional catalyst technologies, reactor configurations, and industrial facilities, will further evaluate the generality of the proposed architecture-dependent transferability framework. Despite these limitations, the results provide practical guidance for industry on selecting the appropriate calibration strategy. Universal models may be considered acceptable only when (i) transferability loss remains within the intrinsic prediction uncertainty of the calibration model, (ii) latent variable structures remain statistically consistent across product groups, and (iii) feature importance rankings preserve their physicochemical interpretation. Conversely, architecture-specific calibration becomes preferable whenever significant degradation in predictive accuracy is accompanied by systematic latent space rotation, changes in dominant process variables, or deterioration in cross-product validation performance. Under these conditions, the polymer architecture should be incorporated as a primary modeling descriptor rather than treated as a secondary process variable. Finally, the present findings suggest that the practical limits of universal MFI prediction arise from the underlying physicochemical mechanisms governing polymer formation rather than solely from algorithmic complexity. Therefore, future improvements in industrial soft sensors are expected to benefit more from architecture-aware modeling strategies than from increasingly sophisticated machine learning algorithms alone.

Although the present results consistently support the proposed hypothesis, the hypothesis remains scientifically falsifiable rather than universally assumed. Specifically, it would be challenged if future industrial datasets demonstrated that a single architecture-independent calibration could simultaneously achieve equivalent predictive accuracy, latent space stability, and cross-family transferability across homopolymer, random copolymer, and impact copolymer production without requiring architecture-specific descriptors or nonlinear partitioning. Likewise, the hypothesis would require revision if identical process variables systematically retained the same physicochemical interpretation and predictive importance across all polypropylene architectures. Therefore, the present work should be interpreted not as establishing an immutable rule, but as providing industrial evidence that the polymer architecture currently represents a dominant physicochemical constraint governing model transferability under the investigated production conditions. Future studies involving additional industrial plants, catalyst technologies, reactor configurations, and broader polypropylene portfolios will ultimately determine the generality and practical limits of this architecture-aware framework.

## 4. Conclusions

This study demonstrates that the transferability of melt flow index (MFI) prediction models across industrial polypropylene grades is strongly constrained by the polymer architecture and cannot be explained by statistical or algorithmic complexity alone. By integrating industrial production data, principal component analysis, partial least squares regression, and machine learning algorithms, the results show that homopolymers, random copolymers, and impact copolymers occupy distinct latent statistical domains. These domains arise from architecture-specific differences in molecular weight regulation, ethylene incorporation, crystallinity, phase morphology, and melt rheological response.

Principal component analysis revealed that increasing the architectural complexity reduces the latent space compressibility. Low-dimensional structures more readily represented homopolymer and random copolymer datasets, whereas impact-rich and full-portfolio datasets required a broader multivariate description. This behavior reflects the transition from monophasic polypropylene systems, where the MFI is mainly controlled by the molecular weight, to compositionally and morphologically complex materials in which comonomer incorporation, rubber-phase formation, and heterophasic morphology contribute additional structure–property pathways.

PLS models provided reliable predictions within architecture-restricted domains but showed reduced predictive efficiency when different polypropylene families were merged into a single calibration space. This loss of performance indicates that a universal linear latent variable model cannot fully preserve the distinct process–structure–MFI relationships present in industrial polypropylene portfolios. Machine learning models, particularly tree-based ensemble methods such as Extra Trees, Random Forest, and Gradient Boosting, improved within-domain prediction by capturing nonlinear interactions among hydrogen-related descriptors, catalyst and donor variables, reactor conditions, productivity, and architecture-specific process variables. However, product group cross-validation showed that improved within-pool accuracy does not necessarily imply robust cross-family transferability.

The combined results support a physicochemical interpretation in which the MFI is not a simple function of the molecular weight alone. Instead, the MFI represents the macroscopic expression of a hierarchical structure–property cascade linking the reactor operating conditions, polymer architecture, molecular descriptors, phase morphology, and melt rheology. Hydrogen-mediated chain transfer remains the dominant molecular weight control mechanism. Still, its predictive role is modulated by ethylene incorporation in random copolymers and by rubber-phase morphology and second reactor effects in impact copolymers. Consequently, the same industrial process variable can have different statistical and physicochemical meanings depending on the polypropylene architecture. The principal contribution of this work is the demonstration that the polypropylene architecture is not merely a categorical production label but a physicochemical state variable that governs latent space organization, model complexity, and predictive transferability. In this sense, the architecture links reactor operation with the molecular structure, morphology, melt rheology, and ultimately the MFI. This interpretation provides a mechanistic basis for deciding when a universal soft sensor is acceptable and when architecture-specific or architecture-aware calibration is required. Accordingly, machine learning algorithms should be regarded primarily as tools for identifying statistically relevant process–structure relationships rather than as mechanistic models by themselves. Their industrial value increases substantially when their predictions are interpreted within the established framework of the polymerization kinetics, molecular architecture, phase morphology, and melt rheology. In this sense, data-driven modeling complements, rather than replaces, physicochemical understanding.

Overall, this work establishes the polymer architecture as a fundamental descriptor for industrial MFI modeling. Architecture-specific or architecture-routed predictive models provide a more physically interpretable strategy than architecture-blind universal calibration when cross-family validation deteriorates. The present steady-state framework informs the future development of chemometric soft sensors and physics-informed digital twins, but extension to grade transitions requires a separate dynamic validation program.

This study therefore supports a shift from purely algorithm-centered soft sensor development toward physicochemically informed machine learning, where statistical learning and polymer science jointly define model design, validation, and industrial applicability.

## Figures and Tables

**Table 1:** Physicochemical characteristics governing melt flow generation in the reactor-grade polypropylene architectures investigated in this study.

Property	Homopolymer	Random Copolymer	Impact Copolymer	Influence on MFI
Polymerization configuration	Single gas-phase reactor	Single gas-phase reactor with ethylene feed	Sequential two-reactor gas-phase process	Very High
Main monomer(s)	Propylene	Propylene + ethylene	Propylene + ethylene–propylene rubber	High
Polymer architecture	Isotactic PP	Random propylene–ethylene copolymer	Heterophasic polypropylene	Very High
Dominant chain transfer mechanism	Hydrogen-mediated	Hydrogen + comonomer incorporation	Hydrogen + dual-reactor kinetics	High
Ethylene incorporation	Absent	Randomly distributed along PP chains	Concentrated mainly in the rubber phase	High
Molecular weight regulation	Mainly hydrogen	Hydrogen + comonomer effects	Hydrogen + matrix/rubber interaction	High
Molecular weight distribution	Relatively narrow	Intermediate	Broad and multimodal	High
Crystallinity	High	Intermediate	Low–intermediate	Medium
Morphology	Monophasic	Modified monophasic	Heterophasic	Very High
Rubber phase	Absent	Absent	Present	Very High
Dominant rheological mechanism	Molecular weight controlled	Molecular weight + chain irregularity	Matrix/rubber interaction	Very High
Primary physicochemical origin of MFI	Molecular weight reduction	Molecular weight + crystallinity	Matrix molecular weight + rubber morphology	Very High

**Table 2:** Summary of principal component analysis for the industrial polypropylene datasets.

Dataset	PP Architecture	Observations	Predictors	PC1 Variance (%)	PC2 Variance (%)	Cumulative Variance PC1+PC2 (%)
H-05H82	Homopolymer	27	26	38.68	24.93	63.60
R-MF(2–5)	Random copolymer	21	27	52.34	22.90	75.24
Impact	Impact copolymer	110	40	27.09	10.85	37.94
R+I	Random + impact copolymers	147	40	27.32	14.77	42.09
H+R+I	Full polypropylene portfolio	425	26	28.92	17.56	46.48

**Table 3:** Predictive performance of the PLS models.

Dataset	PP Architecture	*n*	Optimal Latent Variables	CV-R^2^ log10(MFI)	RMSE log10(MFI)	MAE log10(MFI)
H-05H82	Homopolymer	27	5	0.799	0.045	0.032
R-MF(2–5)	Random copolymer	21	1	0.740	0.023	0.016
Impact	Impact copolymer	110	8	0.857	0.189	0.134
R+I	Random + impact copolymers	147	8	0.714	0.303	0.206
H+R+I	Full polypropylene portfolio	425	4	0.606	0.225	0.161

**Table 4:** Best-performing machine learning models for MFI prediction.

Dataset	PP Architecture	Best Model	CV-R^2^ log10(MFI)	RMSE log10(MFI)	MAE log10(MFI)
**H-05H82**	Homopolymer	PLS	0.767	0.048	0.032
**R-MF(2–5)**	Random copolymer	Random Forest	0.689	0.025	0.019
**Impact**	Impact copolymer	Extra Trees	0.893	0.163	0.094
**R+I**	Random + impact copolymers	Extra Trees	0.952	0.125	0.081
**H+R+I**	Full polypropylene portfolio	Gradient Boosting	0.857	0.136	0.090

**Table 5:** Cross-family transferability metrics of the best-performing predictive models.

Dataset	Best Model	Within-Pool CV-R^2^	Product Group CV-R^2^	Transferability Loss
Random copolymer	Random Forest	0.689	0.350	0.339
Impact copolymer	Extra Trees	0.893	0.834	0.059
Random + impact copolymers	Extra Trees	0.952	0.572	0.380
Full polypropylene portfolio	Gradient Boosting	0.857	0.579	0.278

**Table 6:** Relationship between polypropylene architecture, physicochemical complexity, and model transferability behavior.

Descriptor	Homopolymer	Random Copolymer	Impact Copolymer	Random + Impact	Full Portfolio
Main architecture	Isotactic polypropylene	Poly(propylene-co-ethylene)	Heterophasic PP/EPR	Random and impact copolymers	Homopolymer, random, and impact copolymers
Ethylene incorporation	Absent	Low to moderate	High in rubber phase	Present in both domains	Architecture-dependent
Morphology	Monophasic	Modified monophasic	Heterophasic	Multidomain	Maximum heterogeneity
Crystallinity	High	Intermediate	Low to intermediate	Variable	Broadest range
Dominant MFI mechanism	Molecular weight control	Molecular weight + comonomer effect	Matrix molecular weight + rubber-phase contribution	Shared ethylene-containing descriptors	Multiple structure–property pathways
Main process descriptors	Hydrogen concentration; H2/C3 ratio; catalyst feed	Hydrogen concentration; H2/C3 ratio; ethylene-related variables	Hydrogen concentration; H2/C3 ratio; second reactor and bed descriptors	Hydrogen, ethylene, catalyst, and second reactor descriptors	Combined hydrogen, catalyst, donor, pressure, productivity, and architecture-dependent descriptors
Expected model behavior	Linear latent response	Compact latent response	Nonlinear response	Strong ML improvement	High within-pool accuracy but reduced transferability
Best model	PLS	Random Forest	Extra Trees	Extra Trees	Gradient Boosting
Best CV-R^2^	0.767	0.689	0.893	0.952	0.857
Transferability behavior	Not evaluated	Low cross-family transferability	High within-domain transferability	Strong within-pool fit but high transferability loss	Intermediate transferability

**Table 7:** Process–structure–property relationships explaining architecture-dependent MFI model transferability.

Reactor Variable	Primary Physicochemical Effect	Homopolymer	Random Copolymer	Impact Copolymer	Consequence for Predictive Modeling
Hydrogen concentration	Chain transfer	Dominant	Dominant	Important	Architecture-dependent regression coefficients
C_2_/C_3_ ratio	Ethylene incorporation	Not applicable	Controls chain irregularity	Controls rubber composition	Variable importance changes
Catalyst feed	Active site population	High	High	High	Common predictor
TEAL/Ti ratio	Catalyst activation	High	High	High	Common predictor
External donor	Stereoregularity	Moderate	Moderate	Moderate	Latent space rotation
Reactor temperature	Polymerization kinetics	Moderate	Moderate	Moderate	Nonlinear interactions
Residence time	Molecular growth	Moderate	Moderate	High	Different latent dimensionality
Second reactor variables	Rubber-phase generation	Absent	Absent	Dominant	Architecture-specific predictors
Production rate	Residence time and heat transfer	Moderate	Moderate	Moderate	Shared industrial descriptor
Bed inventory	Fluidization behavior	Moderate	Moderate	High	Architecture-dependent response

## Data Availability

The original contributions presented in this study are included in the article/Supplementary Materials. Further inquiries can be directed to the corresponding author.

## Supplementary Materials

The following supporting information can be downloaded at: https://www.mdpi.com/article/10.3390/polym18151880/s1, Table S1. Industrial datasets used for model development and validation; Table S2. Process variables retained for chemometric and machine-learning modeling; Table S3. Hyperparameter search space used for model optimization; Table S4. Complete machine-learning benchmarking matrix based on cross-validated R2; Table S5. Prediction uncertainty estimated from repeated 5-fold cross-validation; Table S6. Optimized hyperparameters selected for the final Random Forest, Extra Trees, and Gradient Boosting models; Figure S1. Robustness of architecture-aware predictive models under repeated cross-validation; Figure S2. Architecture-dependent latent-space organization in the complete industrial polypropylene portfolio; Figure S3. Effect of the log_10_ transformation on the homoscedasticity of industrial melt flow index data; Figure S4. Predictive robustness against preprocessing choices confirms the stability of the proposed modeling framework.
