# Supplementary material for: Reactor-Grade Non-Additive Polypropylene Architecture Defines the Physicochemical Limits of Melt Flow Index Model Transferability: An Industrial Chemometric and Machine Learning Study
Source: Polymers (Basel). 2026 Jul 31;18(15):1880. doi: 10.3390/polym18151880 (PMC13467857; doi:10.3390/polym18151880)
Supplement: Supplementary file 1 [file polymers-18-01880-s001.zip › polymers-4433429-supplementary.pdf]

Supplementary Materials

Architecture-Aware Transferability of Melt Flow Index Prediction Models in Industrial Polypropylene Portfolios

Table S1. Industrial datasets used for model development and validation.

Dataset	Polypropylene architecture	Commercial grades/families	Production runs	Predictors after preprocessing	Response variable	Modeling purpose
H-05H82	Homopolymer	1 family	27	26	log10(MFI)	Architecture-restricted homopolymer baseline
R-MF(2–5)	Random copolymer	5 grades	21	27	log10(MFI)	Monophasic comonomer-containing architecture
Impact	Impact copolymer	11 grades	110	40	log10(MFI)	Heterophasic architecture-specific model
R+I	Random + impact copolymers	18 grades	147	40	log10(MFI)	Intermediate multi-architecture model
H+R+I	Full polypropylene portfolio	41 grades	425	26	log10(MFI)	Universal portfolio model and transferability evaluation

Table S2. Process variables retained for chemometric and machine-learning modeling.

Variable group	Process variable	Physicochemical or operational meaning	Relevance
Molecular-weight control	Hydrogen concentration	Chain-transfer control and molecular-weight regulation	All datasets
Molecular-weight control	H2/C3 ratio	Relative hydrogen-to-propylene	All datasets

		availability for chain transfer	
Molecular-weight control	H2 feed	Operational hydrogen dosing variable	All datasets
Catalyst-related descriptors	Catalyst feed	Active-site concentration and polymerization rate	All datasets
Catalyst-related descriptors	TEAL flow	Cocatalyst availability and catalyst activation	All datasets
Catalyst-related descriptors	TEAL/SCA ratio	Balance between cocatalyst and selectivity control agent	All datasets
Catalyst-related descriptors	SCA feed	Donor/selectivity control and stereoregularity modulation	All datasets
Catalyst-related descriptors	Donor-to-catalyst ratio	Catalyst-site regulation and donor selectivity	All datasets
Monomer/comonomer descriptors	Propylene concentration	Main monomer availability in the gas phase	All datasets
Monomer/comonomer descriptors	C3PP	Propylene-related process descriptor	All datasets
Monomer/comonomer descriptors	Ethylene concentration	Comonomer incorporation and rubber-phase formation	Random and impact copolymers
Monomer/comonomer descriptors	C2PP	Ethylene-related process descriptor	Random and impact copolymers
Monomer/comonomer descriptors	C2/C3 ratio	Relative ethylene/propylene availability	Random and impact copolymers

Thermal descriptors	Control temperature	Polymerization temperature and kinetic control	All datasets
Thermal descriptors	Bed-dew temperature	Condensed-mode operation and heat-removal behavior	Impact and full portfolio
Reactor-operation descriptors	Reactor pressure	Gas-phase composition, monomer availability, and reactor stability	All datasets
Hydrodynamic descriptors	Cycle gas flow	Fluidization and heat-removal capacity	All datasets
Hydrodynamic descriptors	Bed level	Reactor inventory and fluidized-bed stability	All datasets
Hydrodynamic descriptors	Bed weight	Polymer inventory and residence-time-related behavior	All datasets
Hydrodynamic descriptors	UFBD	Upper fluidized-bed density descriptor	Impact and full portfolio
Hydrodynamic descriptors	LFBD	Lower fluidized-bed density descriptor	Impact and full portfolio
Hydrodynamic descriptors	Condensed-mode operation	Reactor heat-transfer and operation regime	Impact and full portfolio
Productivity descriptors	Productivity	Catalyst productivity and production efficiency	All datasets
Productivity descriptors	Production rate	Overall polymer production rate	All datasets
Residence-time descriptors	Residence time	Polymer residence time and conversion history	All datasets

Second-reactor descriptors	Reactor-2 production rate	Rubber-phase formation and heterophasic architecture control	Impact and R+I datasets
Second-reactor descriptors	Flow to reactor 2	Feed distribution and second-reactor operating regime	Impact and R+I datasets

Table S3. Hyperparameter search space used for model optimization.

Model	Hyperparameter	Search space
PLS	Number of latent variables	1–10
PCR-Ridge	Number of principal components	1–15
PCR-Ridge	Ridge regularization strength	0.001, 0.01, 0.1, 1, 10, 100
Random Forest	Number of trees	300, 500, 1000
Random Forest	Maximum depth	None, 3, 5, 10
Random Forest	Minimum samples per leaf	1, 2, 4
Random Forest	Maximum features	Square root, log2, 0.5, 1.0
Extra Trees	Number of trees	300, 500, 1000
Extra Trees	Maximum depth	None, 3, 5, 10
Extra Trees	Minimum samples per leaf	1, 2, 4
Extra Trees	Maximum features	Square root, log2, 0.5, 1.0
Gradient Boosting	Number of estimators	100, 300, 500
Gradient Boosting	Learning rate	0.01, 0.05, 0.1
Gradient Boosting	Maximum depth	2, 3, 4
Gradient Boosting	Subsample fraction	0.7, 0.9, 1.0
Gradient Boosting	Minimum samples per leaf	1, 2, 4

Table S4. Complete machine-learning benchmarking matrix based on cross-validated R2.

Dataset	PLS	PCR-Ridge	Random Forest	Extra Trees	Gradient Boosting	Best model
H-05H82	0.76732	0.39287	0.39178	0.55091	0.43073	PLS
R-MF(2–5)	-32.84488	-6.18755	0.68887	0.59698	0.63492	Random Forest

Impact	0.84375	0.65393	0.85870	0.89284	0.85920	Extra Trees
R+I	0.59460	0.56843	0.92406	0.95169	0.92639	Extra Trees
H+R+I	0.52519	0.35614	0.85592	0.84394	0.85686	Gradient Boosting

Negative CV-R2 values indicate that the corresponding model performed worse than the mean-response baseline under the cross-validation protocol.

Table S5. Prediction uncertainty estimated from repeated 5-fold cross-validation.

Dataset	Architecture	n	Predictors	Best model	Mean CV-R^2	SD R^2	Mean RMSE	SD RMSE	95% CI RMSE	Mean MAE
H-05H82	Homopolymer	27	26	PLS	0.736	0.103	0.0507	0.0093	0.0449–0.0565	0.0355
R-MF(2–5)	Random copolymer	21	27	Random Forest	0.679	0.064	0.0251	0.0025	0.0236–0.0266	0.0205
Impact	Impact copolymer	110	40	Extra Trees	0.919	0.011	0.1415	0.0093	0.1357–0.1473	0.0986
R+I	Random + impact	147	40	Extra Trees	0.918	0.008	0.1625	0.0080	0.1576–0.1675	0.1104
H+R+I	Full portfolio	425	26	Gradient Boosting	0.870	0.006	0.1294	0.0029	0.1276–0.1311	0.0823

Table S6. Optimized hyperparameters selected for the final Random Forest, Extra Trees, and Gradient Boosting models.

**Model**	**Optimized hyperparameters**	**Search space**	**Validation strategy**	**Selection criterion**	**Application**
Random Forest	Number of trees, maximum depth, minimum samples per leaf, and maximum features	Reported in Table S3	Five-fold cross-validation	Highest mean CV-R^2	Independently optimized within each modeling pool
Extra Trees	Number of trees, maximum depth, minimum samples per leaf, and maximum features	Reported in Table S3	Five-fold cross-validation	Highest mean CV-R^2	Independently optimized within each modeling pool
Gradient Boosting	Number of estimators, learning rate, maximum depth, subsample fraction, and minimum samples per leaf	Reported in Table S3	Five-fold cross-validation	Highest mean CV-R^2	Independently optimized within each modeling pool

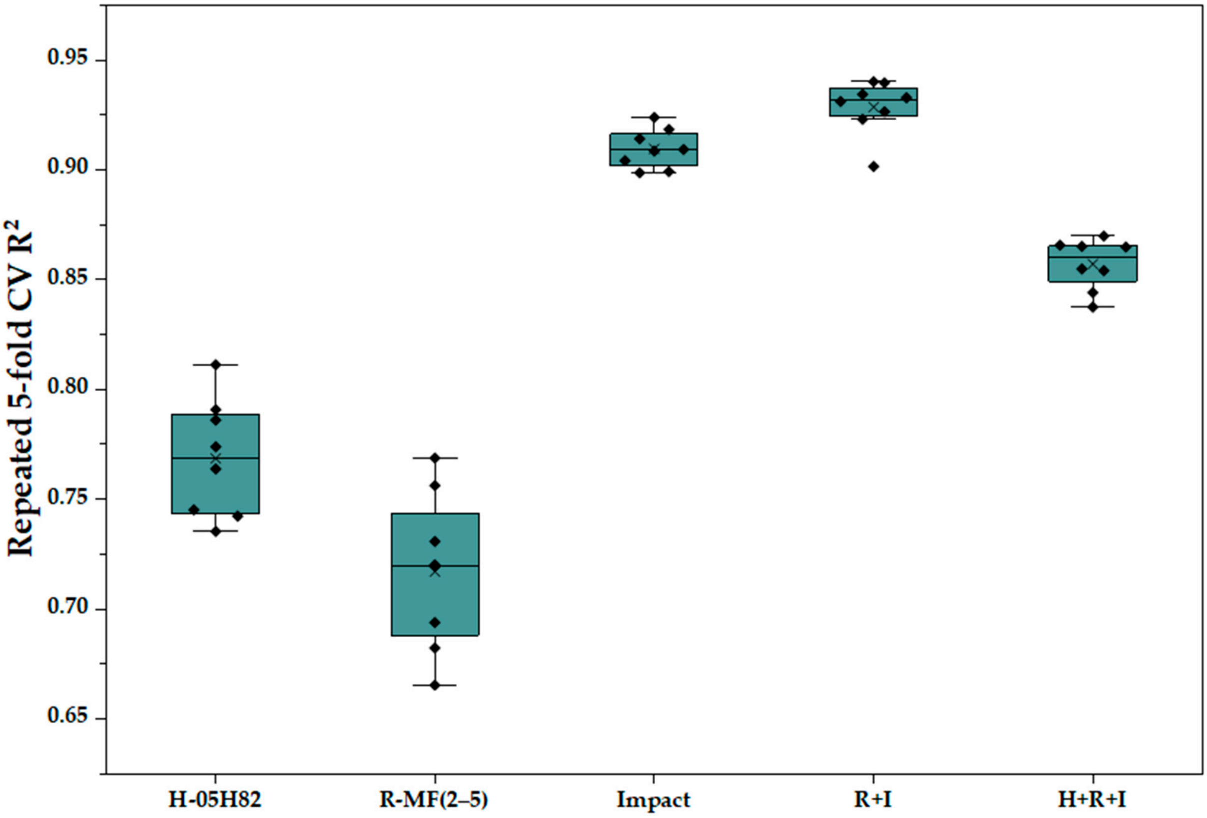

Figure S1. Robustness of architecture-aware predictive models under repeated cross-validation.

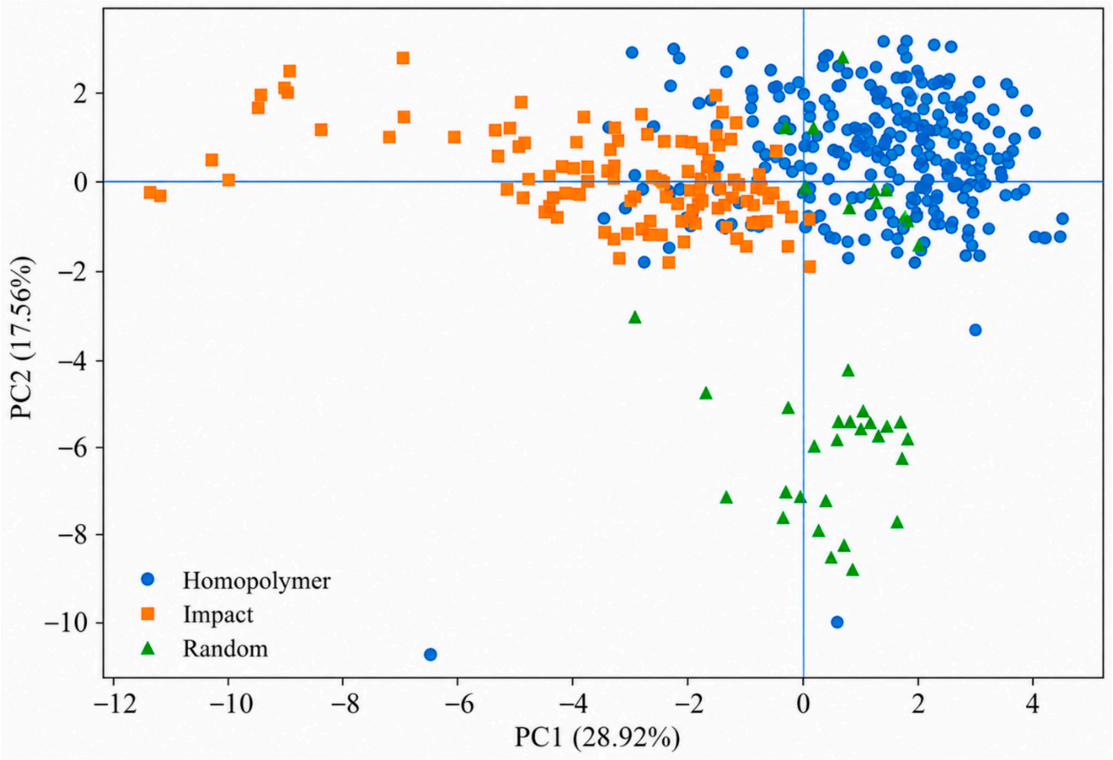

Figure S2. Architecture-dependent latent-space organization in the complete industrial polypropylene portfolio..

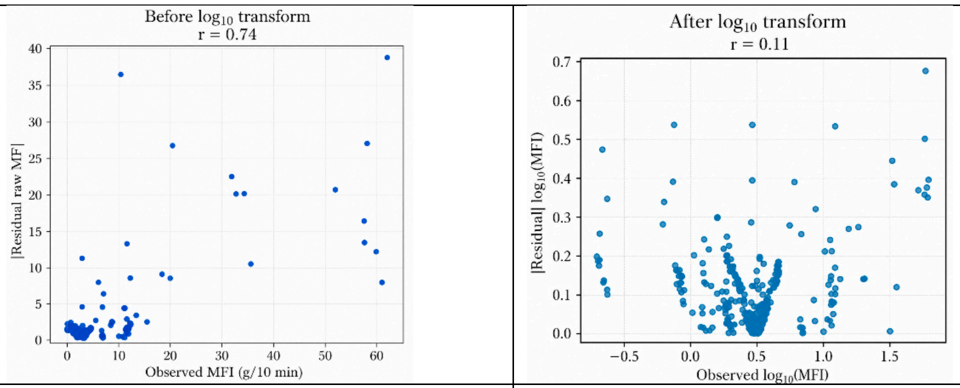

a)	b)

Figure S3. Effect of the $\log_{10}$ transformation on the homoscedasticity of industrial melt flow index data. Absolute residuals are plotted against the observed MFI values before transformation (a) and against the observed $\log_{10}$(MFI) values after transformation (b). The Pearson correlation between residual magnitude and response level decreases from

$r = 0.74$to $r = 0.11$, indicating a substantial reduction in heteroscedasticity and a more uniform residual distribution across the industrial operating range.

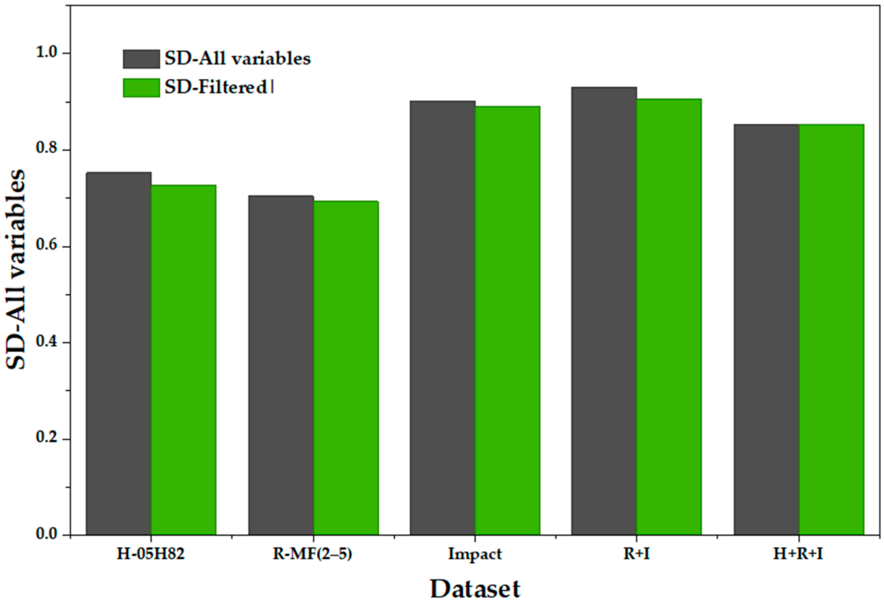

Figure S4. Predictive robustness against preprocessing choices confirms the stability of the proposed modeling framework.
